# Blood biomarkers associated to complete pathological response on NSCLC patients treated with neoadjuvant chemoimmunotherapy included in NADIM clinical trial

**DOI:** 10.1002/ctm2.491

**Published:** 2021-07-19

**Authors:** Raquel Laza‐Briviesca, Alberto Cruz‐Bermúdez, Ernest Nadal, Amelia Insa, María del Rosario García‐Campelo, Gerardo Huidobro, Manuel Dómine, Margarita Majem, Delvys Rodríguez‐Abreu, Alex Martínez‐Martí, Javier De Castro Carpeño, Manuel Cobo, Guillermo López Vivanco, Edel Del Barco, Reyes Bernabé Caro, Nuria Viñolas, Isidoro Barneto Aranda, Santiago Viteri, Bartomeu Massuti, Marta Casarrubios, Belén Sierra‐Rodero, Carlos Tarín, Aránzazu García‐Grande, Cara Haymaker, Ignacio I. Wistuba, Atocha Romero, Fernando Franco, Mariano Provencio

**Affiliations:** ^1^ Servicio de Oncología Médica, Instituto de Investigación Sanitaria Puerta de Hierro‐Segovia de Arana (IDIPHISA) Hospital Universitario Puerta de Hierro‐Majadahonda Madrid Spain; ^2^ Doctoral School Universidad Autónoma de Madrid Madrid Spain; ^3^ Institut Català d'Oncologia, L'Hospitalet De Llobregat Barcelona Spain; ^4^ Fundación INCLIVA Hospital Clínico Universitario de Valencia Valencia Spain; ^5^ Hospital Universitario A Coruña A Coruña Spain; ^6^ Hospital Universitario de Vigo Pontevedra Spain; ^7^ Hospital Universitario Fundación Jiménez Díaz Madrid Spain; ^8^ Hospital de la Santa Creu i Sant Pau Barcelona Spain; ^9^ Hospital Insular de Gran Canaria Las Palmas Spain; ^10^ Hospital Universitario e Instituto de Oncología Vall d´Hebron (VHIO) Barcelona Spain; ^11^ Hospital Universitario La Paz Madrid Spain; ^12^ Hospital Universitario Regional de Málaga Málaga Spain; ^13^ Hospital Universitario Cruces Barakaldo Spain; ^14^ Hospital Universitario de Salamanca Salamanca Spain; ^15^ Hospital Universitario Virgen del Rocio Seville Spain; ^16^ Hospital Clínic Barcelona Spain; ^17^ Hospital Universitario Reina Sofia Córdoba Spain; ^18^ Instituto Oncológico Dr. Rosell, Hospital Universitario Quiron Dexeus Grupo QuironSalud Barcelona Spain; ^19^ Hospital General de Alicante Alicante Spain; ^20^ Bioinformatics Unit Instituto de Investigación Sanitaria Puerta de Hierro‐Segovia de Arana Madrid Spain; ^21^ Flow Cytometry Core Facility Instituto de Investigación Sanitaria Puerta de Hierro‐Segovia de Arana (IDIPHISA) Madrid Spain; ^22^ Departments of Translational Molecular Pathology The University of Texas MD Anderson Cancer Center Houston Texas USA

**Keywords:** biomarkers, chemoimmunotherapy, immune cells, neoadjuvant, non‐small cell lung cancer

## Abstract

**Background:**

Immunotherapy is being tested in early‐stage non‐small cell lung cancer (NSCLC), and achieving higher rates of complete pathological responses (CPR) as compared to standard of care. Early identification of CPR patients has vital clinical implications. In this study, we focused on basal peripheral immune cells and their treatment‐related changes to find biomarkers associated to CPR.

**Methods:**

Blood from 29 stage IIIA NSCLC patients participating in the NADIM trial (NCT03081689) was collected at diagnosis and post neoadjuvant treatment. More than 400 parameters of peripheral blood mononuclear cells (PBMCs) phenotype and plasma soluble factors were analyzed.

**Results:**

Neoadjuvant chemoimmunotherapy altered more than 150 immune parameters. At diagnosis, 11 biomarkers associated to CPR were described, with an area under the ROC curve >0.70 and *p*‐value <.05. CPR patients had significantly higher levels of CD4^+^PD‐1^+^ cells, NKG2D, and CD56 expression on T CD56 cells, intensity of CD25 expression on CD4^+^CD25hi^+^ cells and CD69 expression on intermediate monocytes; but lower levels of CD3^+^CD56^–^CTLA‐4^+^ cells, CD14^++^CD16^+^CTLA‐4^+^ cells, CTLA‐4 expression on T CD56 cells and lower levels of b‐NGF, NT‐3, and VEGF‐D in plasma compared to non‐CPR. Post treatment, CPR patients had significantly higher levels of CD19 expression on B cells, BCMA, 4‐1BB, MCSF, and PARC and lower levels of MPIF‐1 and Flt‐3L in plasma compared to non‐CPR.

**Conclusions:**

Patients achieving CPR seem to have a distinctive peripheral blood immune status at diagnosis, even showing different immune response to treatment. These results reinforce the different biology behind CPR and non‐CPR responses.

## Graphical abstract

1. Non‐small cell lung cancer (NSCLC) patients achieving complete pathologic response (CPR) after neoadjuvant chemoimmunotherapy seem to have a distinctive peripheral blood immune status at diagnosis and surgery.

2. At diagnosis, CPR patients are characterized by a stronger previously induced immune response with a higher cytotoxic profile and lower levels of inhibitory cytokines and cells.

3. This exploratory analysis of the NADIM study supports the use of blood as a valid source for response biomarkers and may serve as a first step to elucidate response mechanisms to chemoimmunotherapy in NSCLC.

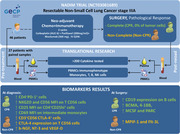

## 1 INTRODUCTION

Non‐small cell lung cancer (NSCLC) accounts for 80%–85% of all lung cancer cases, being the primary cause of cancer‐related death worldwide and the second by incidence.^1^ Over 30% of NSCLC patients are diagnosed at locally advanced stage known as stage III.^2^ Specifically, patients with resectable stage III disease, which are potentially curable, are usually treated with multimodal neoadjuvant treatment, based on chemotherapy and surgery.^3^ However, clinical outcomes of current treatments based on chemotherapy are still discouraging, with complete pathological responses (CPR) near 4%,^4^ and 2‐year overall survival (OS) rates of 60%.^5^

Cancer immunology knowledge has remarkably advanced with the discovery of immune checkpoint proteins. The immune checkpoint PD‐1 in lymphocytes^6^ and myeloid cells^7^ binds to PD‐L1 in the tumor cells, leading to immune cell inactivation and tumor escape.^8^ This has led to anti‐PD‐1 or anti‐PD‐L1 antibodies use against cancer, bringing a new era of treatments known as immunotherapies.^9^ The adaptive immune response lead by T cells plays a major role on antitumor responses^10^; however, macrophages,^11^ natural killer (NK) cells,^12^ B cells,^13^, ^14^ or NK‐T cells^15^ are also involved in antitumor responses.

Immunotherapy treatment has become the standard of treatment for advanced stages and is now being studied for early and locally advanced stages.^16^ Recently, the combination of nivolumab (anti‐PD‐1) and platinum‐based chemotherapy as neoadjuvant treatment for resectable stage III (NADIM clinical trial, NCT03081689) has shown 63% of CPR for resected patients, and 2‐year OS of 90%.^17^ These results open the possibility of transforming locally advanced NSCLC to a curable disease in a substantial percentage of patients, as CPR may be predictive of long‐term survival following neoadjuvant therapy.^18^, ^19^

Consequently, there is a clinical need for predictive biomarkers to identify such patients achieving CPR, which could potentially be free of disease after neoadjuvant treatment. Current biomarkers such as PD‐L1 tumor proportion score (TPS) and Tumor mutational burden (TMB) remain controversial in clinical practice for anti‐PD‐1 monotherapies.^20^, ^21^ Moreover, their value for combined chemoimmunotherapy is more limited,^22^ and its power to identify patients achieving CPR insufficient.^17^, ^23^

Thus, additional predictive biomarkers beyond PD‐L1 and TMB are required. In this way, an increasing number of investigations are focusing on the host antitumor immune response. Accordingly, patients achieving CPR showed a distinct tumor infiltrating lymphocytes (TILs) component at surgery evaluation.^17^ Additionally, an association between TILs^24^ or peripheral blood mononuclear cells (PBMCs) characteristics,^25^, ^26^ with clinical benefit of lung cancer patients under immunotherapy, has been described. These differences seem to indicate that CPR may also be reflected in patient's immune system at the peripheral level. This would allow a noninvasive alternative to tissue biopsy in predicting and monitoring treatment outcomes.

Here, we present the first research study focused on the composition and phenotypic characteristics of different peripheral immune cell subpopulations and plasma factors in NSCLC patients receiving chemoimmune neoadjuvant therapy, in order to characterize treatment effectiveness and identify biomarkers of CPR.

## 2 MATERIALS AND METHODS

### 2.1 Patients and samples

Patients were selected from NADIM clinical trial: neoadjuvant chemotherapy plus nivolumab in resectable stage IIIA non‐small‐cell lung cancer, an open‐label, multicenter, single‐arm, phase 2 trial.^17^ All patients with available peripheral blood sample were included. Peripheral blood samples from 30 patients at baseline (pretreatment) and 34 patients after three cycles of neoadjuvant treatment with nivolumab plus carboplatin (posttreatment) were obtained. Blood samples at both time points were available for 29 patients. Informed consent for the collection of research samples and study protocol were approved by the clinical research ethics committee of Hospital Puerta de Hierro and the Spanish Lung Cancer Group (SLCG) Board in accordance with the International Conference on Harmonization Guidelines on Good Clinical Practice and the Declaration of Helsinki. Clinical characteristics of patients with blood sample available for this study are described in Table S1. Hospitals and patients recruited are listed in Table S2.

Pathologic response evaluation was carried out as previously described following international guidelines recommendations.^17^, ^27^ We classified patients in complete pathological response (CPR, 0% of viable tumor cells in tumor bed or any lymph node tested) and non‐CPR (patients with any percentage of viable tumor cells in resection specimens). Patients that did not undergo surgery were excluded from the pathological response analysis; however, they were included in comparisons of treatment effect and correlation analysis. Additionally, PD‐L1 TPS and TMB were retrieved from previous report^17^ and correlated with blood parameters analyzed at diagnosis from this study.

### 2.2 Cell and plasma isolation

Peripheral blood was diluted 1:1 in 1640 RPMI (Corning, NY, USA), and mononuclear cells (PBMCs) were isolated by Lymphoprep (Stemcell, Vancouver, Canada) density gradient centrifugation and cryopreserved using freezing medium (1:1 RPMI/FBS, Linus, Spain) containing 10% DMSO (Carl Roth, Germany) until use. Plasma fraction was collected after density gradient centrifugation was performed and stored at −80°C until use.

### 2.3 Immunophenotyping of PBMCs

Cryopreserved pretreatment and posttreatment PBMCs were thawed in parallel, washed with 5% FBS in 1× PBS, and surface stained with CD3‐PerCP (clone BW264/56), CD4‐Viogreen (clone REA623), CD8‐APCVio770 (clone BW135/80), CD14‐APC (clone Tük4), CD16‐Viogreen (clone REA423), CD19‐FITC (clone LT19), CD56‐APCVio770 (clone REA196), CD69‐FITC (clone REA824), CD107a‐FITC (clone H4A3), CTLA4‐PE (clone BNI3), PD1‐PEVio770 (clone PD1.3.1.3), NKG2D‐PE (clone REA797), NKp44‐PEVio770 (clone 2.29) from Miltenyi Biotec (Germany) and CD25‐PeCy7 (clone M‐A251) from BD Bioscience (NJ, USA). Detection of PD‐1 using anti‐PD‐1 clone PD1.3.1.3 was almost technically abrogated in posttreatment samples due to PD‐1 binding of nivolumab as previously described.^28^ However, percentage of PD‐1+ cells and PD‐1 MFI in PD‐1+ cells was still analyzed in posttreatment samples to discard possible differences between groups.

Cells were acquired on a MACS Quant 10 cytometer and DAPI (ThermoFisher, CA, USA) staining prior acquisition was used to exclude dead cells during analysis on FlowJo V10 software. The median number of viable cells after freezing, thawing, and staining was 93.6%, IQR 91.2–95.2. A minimum of 100,000 lymphocyte events were recorded. Expression of surface markers was evaluated by percentage of positive cells and median fluorescence intensity (MFI) using fluorescence minus one (FMO) controls. Gating strategy for all panels is shown in Figure S1A–F. Flow cytometry analysis was performed only in paired samples (29 patients).

Immunophenotyping of circulating NK cells (CD3^–^CD56^+^), T cells (CD3^+^CD56^–^), cytotoxic T cells (CD3^+^CD8^+^), helper T cells (CD3^+^CD4^+^), T CD56 cells (CD3^+^CD56^+^), B cells (CD3^–^CD19^+^), and monocytes (CD14^+^) was determined.

### 2.4 Immunoassays for detecting cytokines and soluble factors in plasma

Sixteen key soluble factors from plasma were measured in duplicate using the human immuno‐oncology checkpoint protein panel (Cat. #HCKPMAG‐11K, Millipore, MA, USA) following manufacturer's instructions. Plasma levels of 200 cytokines were measured on the same samples using G‐Series Human Cytokine Antibody Array 4000 (RayBiotech, GA, USA) following manufacturer's instructions. Cytokine array and soluble factor analysis were performed on all samples.

### 2.5 Statistical analysis

Raw data generated from flow cytometry and cytokine analysis is available in Table S3. Nonparametric Wilcoxon signed rank test was used for analysis of treatment variation and Mann–Whitney *U*‐test was performed to determine differences between CPR and non‐CPR patients. Pearson's chi‐square test was used for association between categorical groups (for baseline characteristics with pathological response). We considered area under the curve (AUC) >0.70 with significant *p*‐value as relevant predictive biomarker using receiver operating characteristic (ROC) curve analysis. Relationship between variables was done by Spearman´s correlation considering *R* > ±0.80 as strong linear correlation with significant *p*‐value. For all statistical tests, *p*‐value <.05 was considered statistically significant; *, **, and *** indicated *p*‐values <.05, <.01, and <.001, respectively. For *p*‐values between .05 and .1, the numerical value is included. Because the research was designed as a discovery study, *p*‐values were not adjusted in order to maximize the finding of new biomarkers and the generation of new hypothesis. IBM SPSS Statistics 25 was used for all statistical analyses. All authors had access to the data and certify the accuracy of the results presented.

## 3 RESULTS

### 3.1 Patient characteristics

No statistical differences on age, sex, smoking status, histology, and lymph node involvement were observed according to the degree of pathological response (Table 1). Patients included in this study showed similar clinical characteristics to NADIM trial complete cohort (Table S1).

**TABLE 1:** Baseline characteristics of patients according to pathological response

	**Total (*N* = 27)**	**CPR (*N* = 15)**	**Non‐CPR (*N* = 12)**	***p*‐Value (CPR vs. non‐CPR)**
Age (years)^a^	67 (59–72)	69 (60–70)	64 (57.5–72.5)	.695
Sex^b^				.756
Male	21 (77.8)	12 (80)	9 (75)	
Female	6 (22.2)	3 (20)	3 (25)	
Cigarette‐smoking history^b^				.795
Former smoker (≥1 year)	15 (55.6)	8 (53.3)	7 (58.3)	
Current smoker	12 (44.4)	7 (46.7)	5 (41.7)	
Histology^b^				.115
Adenocarcinoma	15 (55.6)	9 (60)	6 (50)	
Squamous	9 (33.3)	3 (20)	6 (50)	
NOS/undifferenciated	3 (11.1)	3 (20)	0	
Nodes^b^				.984
N0	7 (25.9)	4 (26.7)	3 (25)	
N1	2 (7.4)	1 (6.7)	1 (8.3)	
N2	18 (66.7)	10 (66.7)	8 (66.7)	

*Note*: Age, sex, smoking status, histology, and affected lymph nodes comparison between pathological responses groups (CPR, complete pathological response) and non‐CPR using Pearson´s chi‐square test. *p*‐Value of chi‐square or Kruskall–Wallis when adequate.

^a^Median and interquartile range (IQR).

^b^ *N* (%).

At baseline and prior to surgery, similar proportions of circulating NK cells (*p*‐value .864), T cells (*p*‐value .845), CD56^+^ T cells (*p*‐value .306), B cells (*p*‐value .262), and monocytes (*p*‐value .124) were found between CPR and non‐CPR patients (Figure S2A). However, deepening into their phenotype and activation levels, we found significant differences described below.

### 3.2 Association of immunocheckpoints expressed in PBMCs and pathological response

#### 3.2.1 PD‐1 expression on lymphocytes

**FIGURE 1:**
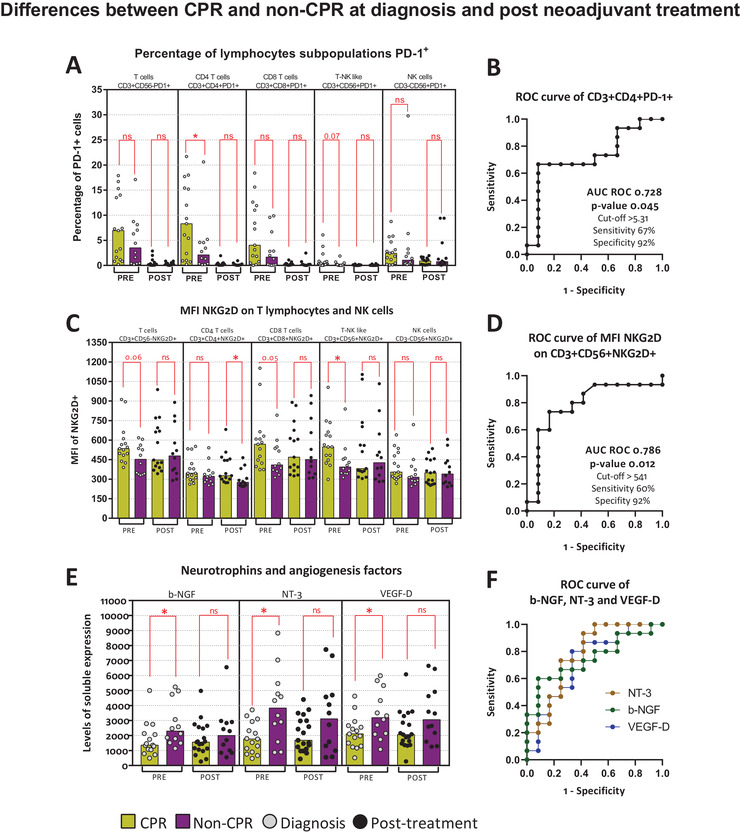
PD‐1 and NKG2D immunocheckpoints and pathological response. Differences between CPR and non‐CPR patients at diagnosis and post neoadjuvant treatment. (A) Percentage of PD‐1^+^ cells on T cells (CD3^+^CD56^–^PD‐1^+^), CD4 T cells (CD3^+^CD4^+^PD‐1^+^), CD8 T cells (CD3^+^CD8^+^PD‐1^+^), T‐NK like (CD3^+^CD56^+^PD‐1^+^) cells, and NK cells (CD3^–^CD56^+^PD‐1^+^). (B) AUC ROC curve for CD3^+^CD4^+^PD‐1^+^. (C) MFI of NKG2D on T cells (CD3^+^CD56^–^NKG2D^+^), CD4 T cells (CD3^+^CD4^+^NKG2D^+^), CD8 T cells (CD3^+^CD8^+^NKG2D^+^), T‐NK like (CD3^+^CD56^+^NKG2D^+^) cells, and NK cells (CD3^–^CD56^+^NKG2D^+^). (D) AUC ROC curve for CD3^+^CD56^+^NKG2D^+^. (E) Levels of b‐NGF, NT‐3, and VEGF‐D in plasma. AUC ROC curve for b‐NGF, NT‐3, VEGF‐D. Legend: Diagnosis (PRE, grey dots), prior to surgery (POST, black dots), CPR patients (yellow bars), and non‐CPR patients (purple bar). Nonparametric Mann–Whitney test was used for comparisons (ns, not significant; *p‐value <.05; **p‐value <.01; ***p‐value <.001) and receiver operating characteristic (ROC) curve analysis showing AUC ROC curve, *p*‐value, and optimum cut‐off

PD‐1 expression and percentages of PD‐1^+^ cells on different lymphocytes subsets, as T cells, CD4^+^ T cells, CD8^+^ T cells, CD56^+^ T cells, and NK cells were measured. At diagnosis, CPR patients showed a higher percentage of PD‐1^+^ cells compared to non‐CPR patients, for all lymphocyte subsets, reaching to statistically significant differences for CD4^+^PD‐1^+^ cells (*p*‐value .045). PD‐1^+^ cells were almost technically undetectable after initiating the treatment with chemotherapy plus nivolumab (Figure 1A). The percentage of CD3^+^CD4^+^PD‐1^+^ cells at diagnosis had a predictive value to distinguish CPR and non‐CPR patients, with an AUC of 0.728 (*p*‐value .045) (Figure 1B). No differences between pathologic response groups were found on MFI PD‐1 expression neither at diagnosis nor after neoadjuvant treatment (Figure S2B).

#### 3.2.2 NKG2D expression on T lymphocytes and NK cells

When analyzing NKG2D expression, we found that at diagnosis, NKG2D expression was higher in CD56^+^ T cells in CPR patients compared to non‐CPR patients (*p*‐value .012) and there was a trend for CD8^+^ T cells (*p*‐value .051) (Figure 1C). Post neoadjuvant treatment, higher expression of NKG2D on CD4^+^ T cells was found in CPR patients (*p*‐value .017) (Figure 1C). NKG2D expression at diagnosis on CD3^+^CD56^+^NKG2D^+^ cells showed an AUC of 0.786 (*p*‐value .012) (Figure 1D).

On the contrary, no significant differences at diagnosis or post treatment in the percentage of NKG2D^+^ cells for T cells, CD56^+^ T cells, and NK cells between CPR and non‐CPR patients were obtained. However, a trend toward higher percentage of CD8^+^NKG2D^+^ cells was observed in CPR patients (*p*‐value .07) (Figure S2C).

Furthermore, we noticed that NKG2D expression inversely correlate with plasma levels of neurotrophin 3 (NT‐3) on CD8 T cells and T CD56 cells, as well as with vascular endothelial growth factor D levels (VEGF‐D) on T CD56 cells (Figure S3A–D). Lower levels of these factors and the nerve growth factor beta (b‐NGF) were found on plasma from CPR patients at diagnosis (Figure 1E). Moreover, these soluble factors can be used as biomarkers associated to CPR at diagnosis, shown by an AUC of 0.750 (*p*‐value .028) for b‐NGF, 0.778 (*p*‐value .015) for NT‐3, and 0.728 (*p*‐value .045) for VEGF‐D (Figure 1F and Figure S3F–H).

#### 3.2.3 CTLA‐4 expression on lymphocytes

**FIGURE 2:**
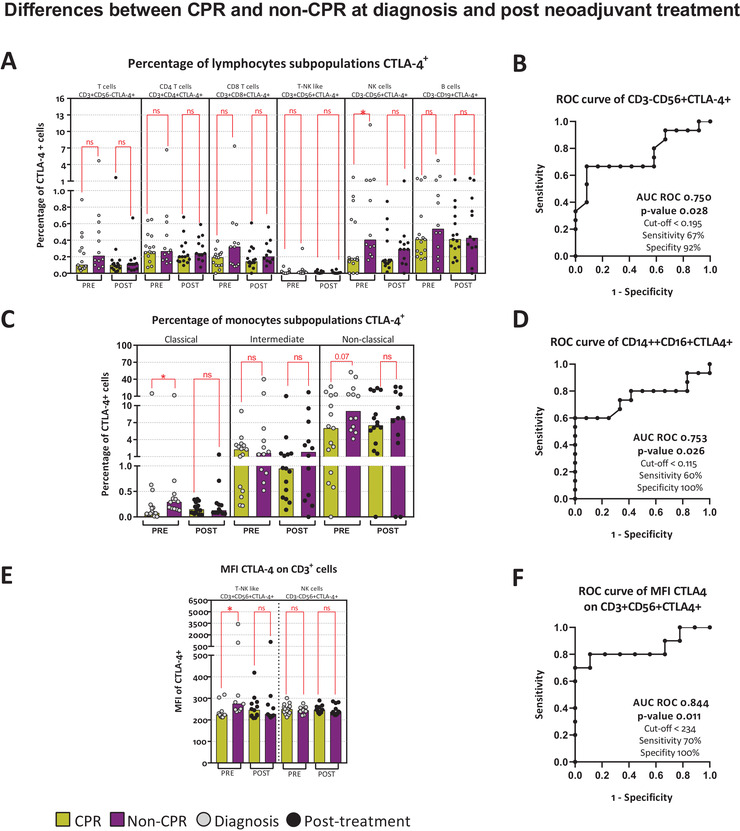
CTLA‐4 immunocheckpoint and pathological response. Differences between CPR and non‐CPR patients at diagnosis and post neoadjuvant treatment. (A) Percentage of CTLA‐4^+^ cells on T cells (CD3^+^CD56^–^CTLA‐4^+^), CD4 T cells (CD3^+^CD4^+^CTLA‐4^+^), CD8 T cells (CD3^+^CD8^+^CTLA‐4^+^), T‐NK like (CD3^+^CD56^+^CTLA‐4^+^) cells, NK cells (CD3^–^CD56^+^CTLA‐4^+^), and B cells (CD3^–^CD19^+^CTLA‐4^+^). (B) AUC ROC curve for CD3^–^CD56^+^CTLA‐4^+^. (C) Percentage of CTLA‐4^+^ cells on classical monocytes (CD14^++^CD16^–^CTLA‐4^+^), intermediate monocytes (CD14^++^CD16^+^CTLA‐4^+^), and nonclassical monocytes (CD14^+^CD16^+^CTLA‐4^+^). (D) AUC ROC curve for CD14^++^CD16^–^CTLA‐4^+^. (E) MFI of CTLA‐4^+^ on T‐NK like cells (CD3^+^CD56^+^CTLA‐4^+^), and T cells (CD3^+^CD56^–^CTLA‐4^+^). (F) AUC ROC curve for CD3^+^CD56^+^CTLA‐4^+^. Legend: Diagnosis (PRE, grey dots), prior to surgery (POST, black dots), CPR patients (yellow bars), and non‐CPR patients (purple bar). Nonparametric Mann–Whitney test was used for comparisons (ns, not significant; *p‐value <.05; **p‐value <.01; ***p‐value <.001) and receiver operating characteristic (ROC) curve analysis showing AUC ROC curve, *p*‐value, and optimum cut‐off

We analyzed CTLA‐4^+^ surface expression on T cells, CD56^+^ T cells, NK cells, B cells, and monocytes and, despite of the scarcity of CTLA‐4^+^ cells in peripheral blood, significant differences were found on NK cells and monocytes (Figure 2). We observed lower pretreatment percentages of CTLA‐4^+^ NK cells (CD3^–^CD56^+^CTLA‐4^+^) in patients with CPR (Figure 2A). Similarly, lower percentages of CTLA‐4^+^ classical monocytes (*p*‐value .026) and nonclassical monocytes (*p*‐value .071) were determined in CPR patients (Figure 2C). At diagnosis, higher expression of CTLA‐4 was found on CD56^+^ T cells (CD3^+^CD56^+^CTLA‐4^+^), but not on T cells (CD3^+^CD56^–^CTLA‐4^+^) in non‐CPR patients (Figure 2E). These three subsets of cells showed an AUC ROC of 0.750 (*p*‐value .028), 0.753 (*p*‐value .026), and 0.844 (*p*‐value .011), respectively (Figure 2B,D,F).

Summarizing these initial results, our study describes the relevance of PD‐1, CTLA‐4, and NKG2D immunocheckpoints on immune cells subsets and, even greater, their value as biomarkers associated to complete pathological response to neoadjuvant chemoimmunotherapy in lung cancer patients.

### 3.3 Association between immune phenotype of PBMCS and pathological response

#### 3.3.1 CD56 expression on NK and T cells

**FIGURE 3:**
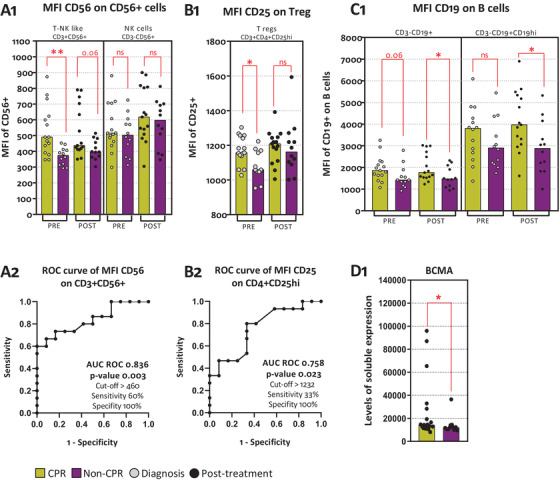
Immunophenotyping associated to pathological response. (A1) MFI of CD56^+^ cells on T‐NK like (CD3^+^CD56^+^) cells and NK cells (CD3^–^CD56^+^). (A2) AUC ROC curve of CD56 MFI on CD3^+^CD56^+^. (B1) MFI of CD25 on CD3^+^CD4^+^CD25^hi^. (B2) AUC ROC curve of CD25 MFI on CD3^+^CD4^+^CD25^hi^. (C1) MFI of CD19 on CD3^–^CD19^+^ and CD3^–^CD19^hi^. (D1) BCMA levels of expression. Legend: Diagnosis (PRE, grey dots), prior to surgery (POST, black dots), CPR patients (yellow bars), and non‐CPR patients (purple bar). Nonparametric Mann–Whitney test was used for comparisons (ns, not significant; *p‐value <.05; **p‐value <.01; ***p‐value <.001) and receiver operating characteristic (ROC) curve analysis showing AUC ROC curve, *p*‐value, and optimum cut‐off

CD56 expression was used as a cytotoxic activation marker in NK and CD56^+^ T cells. CPR patients had higher levels of CD56 expression on CD56^+^ T cells at diagnosis, (*p*‐value .003) and post treatment (*p*‐value .060) (Figure 3A1). Moreover, the AUC of CD56 expression at diagnosis on CD56^+^ T cells was 0.836 (*p*‐value .003). Thus, MFI higher than 460 relative units predicts, with a 60% sensitivity and 100% specificity, patients achieving CPR. This population of patients entails one‐third of analyzed patients (nine out of 27) (Figure 3A2).

We did not find any differences in CD56^+^ T cells and NK cells percentages, even though CPR patients showed a trend toward higher CD56^+^ T cells at diagnosis (*p*‐value .306) and post treatment (*p*‐value .092) (Figure S2D). In addition, CD56 expression on CD56^+^ T cells was inversely correlated to b‐NGF (*p*‐value .031) shown in Figure S3E.

#### 3.3.2 CD25 expression on CD4 T lymphocytes

Regulatory T cells (Tregs) were determined selecting CD4^+^CD25^high^ cells, as described in methods (Figure S1B). At diagnosis, CPR patients had higher levels of CD25 expression on CD3^+^CD4^+^CD25^high^ (Figure 3B1). The value of pretreatment CD25 MFI on regulatory T cells as a biomarker associated to CPR is shown by an ROC AUC of 0.758 (*p*‐value .023) (Figure 3B2). Meanwhile, no differences were seen in percentage of CD3^+^CD4^+^CD25^hi^ cells, at diagnosis or post treatment (Figure S2E).

#### 3.3.3 CD19 expression on B cells

B cells were subdivided, finding a larger size population named as CD19^high^, as described in methods (Figure S1B). Post neoadjuvant treatment, CPR patients have higher levels of CD19 MFI on B cells and on CD19^high^ cells post treatment (Figure 3C1). Furthermore, higher posttreatment levels of TNF receptor superfamily member 17 (TNFRSF17; BCMA) were found on CPR patients (Figure 3D1). On the contrary, no differences were seen in percentage of B cells or CD19^hi^ cells at diagnosis or following neoadjuvant treatment for CPR patients or non‐CPR patients (Figure S2F).

#### 3.3.4 Monocytes and macrophages activation

**FIGURE 4:**
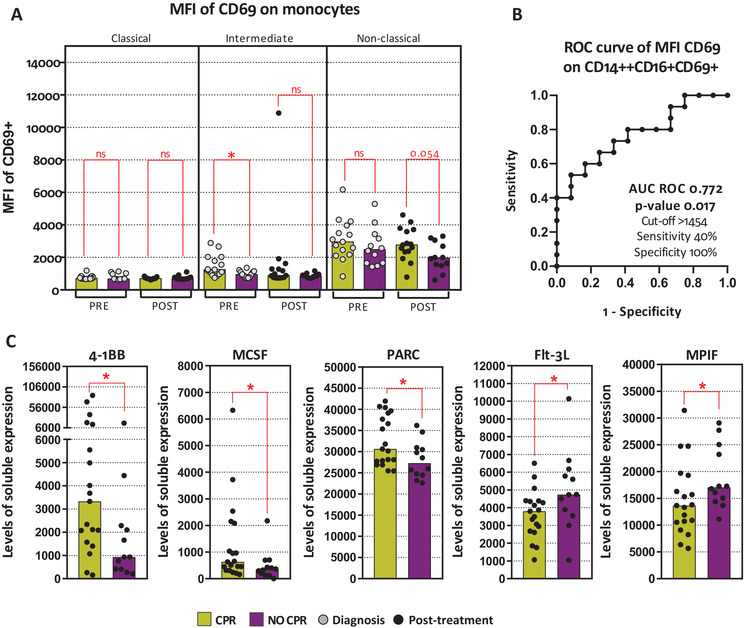
Monocytes and macrophages activation. (A1) MFI of CD69 on classical monocytes (CD14^++^CD16^–^CD69^+^), intermediate monocytes (CD14^++^CD16^+^CD69^+^), and nonclassical monocytes (CD14^+^CD16^+^CD69^+^). (A2) AUC ROC curve of CD69 MFI on CD14^++^CD16^+^CD69^+^. (B) 4‐1BB levels of expression. (C) MCSF levels of expression. (D) PARC levels of expression. (E) Flt‐3L levels of expression. (F) MPIF levels of expression. Legend: Diagnosis (PRE, grey dots), prior to surgery (POST, black dots), CPR patients (yellow bars), and non‐CPR patients (purple bar). Nonparametric Mann–Whitney test was used for comparisons (ns, not significant; *p‐value <.05; **p‐value <.01; ***p‐value <.001) and receiver operating characteristic (ROC) curve analysis showing AUC ROC curve, *p*‐value, and optimum cut‐off

To measure activation of monocytes and macrophages, we used CD69 expression and levels of different soluble factors. At diagnosis, only intermediate monocytes were early activated in CPR patients, showing higher CD69 expression (*p*‐value .017) (Figure 4A). The use of these activated intermediate monocytes as CPR biomarker is shown by an AUC ROC of 0.772 (*p*‐value .017) (Figure 4B).

Moreover, posttreatment cytokine levels related to immune system activation, TNF receptor superfamily member 9 (TNFRSF9; 4‐1BB) and macrophages function such as, macrophage colony‐stimulating factor 1 (CSF‐1; MCSF), C–C motif chemokine ligand 18 (CCL18; PARC), Fms‐related receptor tyrosine kinase 3 ligand (Flt‐3L) and C–C motif chemokine ligand 23 (CCL23; MPIF‐1), seem to have an association with pathological response. We found higher levels of 4‐1BB, MCSF, and PARC but lower levels of Flt‐3L and MPIF‐1 in CPR patients after neoadjuvant treatment (Figure 4C).

### 3.4 Correlations between immune phenotype of PBMCs and cytokines

To unravel potential association between immune cell subpopulations and cytokines, we assessed the correlations among the described biomarkers and pathologic response.

At diagnosis, there is a positive correlation between CD19 expression on B cells with CD4^+^PD‐1^+^ cells (*R* 0.419; *p*‐value .024), CD25 expression on Tregs CD4^+^CD25^high^ (*R* 0.570; *p*‐value .001), and CD56 expression on CD56^+^ T cells (*R* 0.512; *p*‐value .005) (Figure S4A–C). A positive correlation between CD56 expression on CD56^+^ T cells and CD25 expression on Tregs (*R* 0.555; *p*‐value .002) was found (Figure S4D). Furthermore, NKG2D expression on T cells correlate positively with CD56 expression (*R* 0.465; *p*‐value .011) and NKG2D expression (*R* 0.652; *p*‐value .0001) on CD56^+^ T cells (Figure S4E,F).

Following neoadjuvant treatment, we observed a negative correlation between CD19 expression on B cells and NKG2D on T cells (*R* −0.488; *p*‐value .007) and CD56^+^ T cells (*R* −0.565; *p*‐value .001). A positive correlation was found between NKG2D expression on T cells and CD56^+^ T cells (*R* 0.783; *p*‐value .000) (Figure S4G–I).

Nevertheless, focusing on cytokines, on one hand, NT‐3 correlated positively with VEGF‐D and Flt‐3L, and negatively with PARC and MPIF‐1 (Figure S5A–D). Similarly occurred with VEGF‐D, as it correlated positively with b‐NGF and Flt‐3L and negatively with PARC (Figure S5E–G). On the other hand, there was a positive correlation between PARC and Flt‐3L and a negative correlation with MPIF‐1 (Figure S5H,I). Lastly, BCMA positively correlated with 4‐1BB (Figure S5J).

Remarkably, none of the blood parameters analyzed at diagnosis in this study were associated to basal PD‐L1 TPS or TMB (data not shown).

### 3.5 Neoadjuvant treatment influence on immune cells and plasma factors

We also describe the significant effect of chemoimmunotherapy on immune cells (Table S4A,B) and plasma factors (Table S5A–C) in all patients and stratifying by pathologic responses. As previously shown, percentage of general population T cells, CD4^+^ T cells, CD8^+^ T cells, monocytes, B cells, NK cells, and CD56^+^ T cells did not vary with treatment (Figure S2A).

**FIGURE 5:**
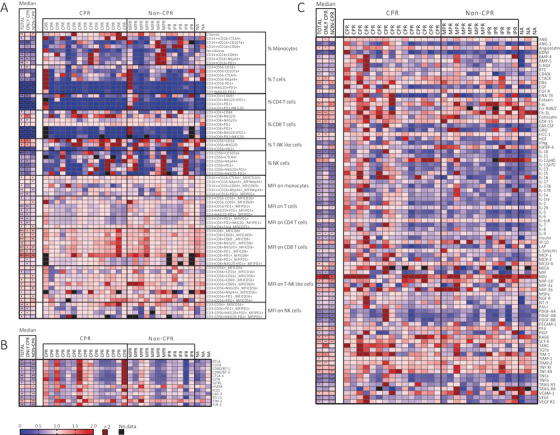
Neoadjuvant treatment influence on immune cells and plasma factors. (A) Percentage of immune cells from peripheral blood and MFI on immune cells from peripheral blood. (B) Soluble factors secretion in plasma. (C) Cytokine secretion on plasma. Legend: Fold‐change (POST/PRE) from 0 to 2, 0 indicating (blue) a decrease after treatment, 1 (pink) indicates no changes, 2 (red) indicates increase, and >2 (dark red) indicates more than double of increase, and black squares (no data). Nonparametric Wilcoxon test was used for comparisons, (empty, not significant; *p‐value <.05; **p‐value <.01; ***p‐value <.001)

Independently of pathologic response and following treatment initiation, a decrease of cells’ percentages was observed for most of the subgroups except for CD8^+^PD‐1^–^, CD8^+^NKG2D^–^, CD3^+^CD56^+^CD16^–^, and CD3^+^CD56^+^NKG2D^–^. Moreover, these cells are increased after treatment only in CPR patients. (Figure 5A).

However, when looking at the expression of analyzed markers by MFI, all patients have a slightly decrease on PD‐1 detection on monocytes, T cells, CD4^+^ T cells, CD8^+^ T cells, and NK cells (Figure 5A). Regarding CD8 expression, measured as MFI, there was an increase on CD8 expression in all patients (*p*‐value .0004). Concerning CD56, on one hand, its expression on NK cells increased after treatment in all patients (*p*‐value .002) and this significance was only maintained in CPR patients (*p*‐value .031). On the other hand, CD56 expression on CD56^+^ T cells was increased only in non‐CPR patients (*p*‐value .019) (Figure 5A).

Focusing on the effect of neoadjuvant chemoimmunotherapy on cytokines and soluble factors, there were no differences found on soluble factors as a predictive biomarker or associated to pathological response. However, when comparing samples at diagnosis versus postneoadjuvant treatment, major differences were found in non‐CPR patients with a decrease, post treatment, of all soluble factors analyzed except for HVEM (*p*‐value .049) and TIM‐3 (*p*‐value .002) (Figure 5B).

Lastly, levels of many cytokines changed after treatment. Considering the focus of this study and space limitation, we describe here only the changes that are exclusive of patients achieving CPR. These patients showed an increase of ANG (*p*‐value .027), angiostatin (*p*‐value .041), CTACK (*p*‐value .031), HCC‐1 (*p*‐value .001), MICA (*p*‐value .041), RAGE (*p*‐value .041), TARC (*p*‐value .020), TIM‐1 (*p*‐value .020), and TIMP‐2 (*p*‐value .003), and a decrease of Fcr RIIB/C (*p*‐value .045), GRO (*p*‐value .036), and TRAIL‐R4 (*p*‐value .041) (Figure 5C).

## 4 DISCUSSION

Immunotherapy alone or in combination with chemotherapy is revolutionizing the therapeutic approach of early‐stage lung cancer patients, achieving a remarkable rate of CPR in potentially resectable patients. In lung cancer, CPR has been proposed as a surrogate of OS benefit in the neoadjuvant setting. In our study, patients achieving CPR showed a trend toward higher basal levels of PD‐1^+^ cells for T, NK, and T subsets, which was statistically significant only for CD4^+^, but not for CD8^+^ T cells.

To our knowledge, there are no studies focused on predictive peripheral biomarkers of complete pathological responses in the neoadjuvant setting. Besides, as chemoimmunotherapy seems superior to monotherapy^16^ and chemotherapy modifies the antitumor immune response,^29^, ^30^ the potential biomarkers described for anti‐PD(L)‐1 monotherapy may be useless for chemoimmunotherapy. In fact, it is likely that these biomarkers may vary also depending on tumor stage, treatment timing (neoadjuvant or adjuvant), and the pathologic response rate used as threshold (CPR vs. non‐CPR analysis identified different parameters compared to MPR vs. non‐MPR in our cohort), making the contrast of our results an arduous task. Additionally, PD‐L1 TPS and TMB at diagnosis were not associated to any parameter analyzed in this study, indicating an independent value of these blood biomarkers.

Although PD‐1 interaction with its ligands leads to T‐cell inhibition, PD‐1 expression is induced after antigen recognition. Thus, PD‐1 levels may reflect chronic activation of antitumor‐specific lymphocytes.^31^ Few studies have described the importance of early proliferation of PD‐1^+^ cells, mainly CD8^+^, after anti‐PD‐1 treatment in NSCLC patients.^32^, ^33^

Another population of T cells that seems to recapitulate the basal immune status of patients achieving CPR is Tregs (CD4^+^CD25^hi^). The levels of these cells are higher in the blood of NSCLC patients compared to healthy individuals,^34^, ^35^ they are implicated on immune self‐tolerance^36^ and have been associated with favorable clinical outcomes to anti‐PD‐1 therapy.^37^ We have previously described in NSCLC patients that tumor‐infiltrating Tregs after chemoimmunotherapy are associated with CPR.^17^ In this study, we describe their predictive value for CPR when measured in peripheral blood at diagnosis. The association between CPRs and basal PD‐1^+^ or Tregs cells may reflect the immunogenicity of the tumor and the tolerance to chronic immune responses.

In addition to T cells, CD56^+^ T cells also appear to be characteristic of the immune system of CPR patients. Reduced blood levels of CD56^+^ T cells has been described on NSCLC^38^ and other solid tumors,^39^, ^40^, ^41^ compared to healthy controls. Although, in a similar way to other groups,^42^ the levels of CD56^+^ T cells were not related with response, but we observed that CPR patients had higher expression of activating receptors, such as NKG2D and CD56. Besides the lack of knowledge in CD56^+^ T cells, and in particular of these receptors in responses to anti‐PD‐1 therapy, CD56^+^ T cells have been described as modulators of the immune system.^43^

Traditionally, CD56 receptor is used to classify between two types of NK cells, CD56^bright^ (cytokine‐producing cells) and CD56^dim^ (cytotoxic cells). However, its expression is also widely accepted as activation marker, associated with potent effector functions, in NK, classical αβ CD8 T cells, and CD56^+^ T cells.^44^ Supporting this, we found a correlation between CD56 and NKG2D for CD56^+^ T cells.

NKG2D is a cytotoxic‐related activation receptor expressed in NK, CD8^+^ T cells, and T CD56 cells. NKG2D ligands (MICA, MICB, and ULBP1‐6) are expressed on stressed or tumor cells, favoring their destruction. Interestingly, a tumor evasion mechanism involves releasing these ligands from the tumor cell surface, inducing receptor internalization on immune cells, and therefore reducing their activation capacity.^45^, ^46^ In our study, patients with CPR showed statistically significant higher levels of NKG2D in CD56^+^ T cells and nearly significant in CD8^+^ T cells, but not for NK cells. Furthermore, the levels of MICA and MICB ligands were similar between CPR and non‐CPR patients, suggesting that this evasion mechanism is not relevant in our cohort of patients. On the other hand, NKG2D levels in CD56 T cells and CD8+ T cells were inversely correlated with NT‐3 plasma levels, which could imply a mechanism of inhibition by this neurotrophin on immune cells.^47^, ^48^ Similarly, an inverse correlation between VEGF‐D and NKG2D was observed, although this was only statistically significant for CD56+ T cells. To our knowledge, there is no specific information on the role of VEGF‐D and NKG2D regulation, although a protumoral role of VEGF‐D in lung cancer has been described.^49^

Other receptor involved in tumor evasion mechanisms is CTLA‐4, originally described on T cells. There are few studies on other cell types, such as monocytes and NK cells, pointing in the same direction of inhibiting immune responses.^50^, ^51^ Supporting this, we have seen that CPR patients presented reduced basal levels of CTLA‐4^+^ on NK and classic monocytes. Despite CTLA‐4 and PD‐1 are inhibitory receptors and markers of antigen experienced cells, in our study these receptors behave in the opposite way, which could be explained by their participation in different pathways in which CTLA‐4 is not affected by nivolumab treatment.^52^

In this way, at diagnosis, antitumor immune status for CPR patients seems to be characterized by a previously induced immune response (PD‐1^+^ cells and Tregs), with a cytotoxic profile (NKG2D and CD56 receptors), and lower levels of inhibitory cytokines and cells (neurotrophins, VEGFD, and CTLA‐4^+^ cells). This intense immune response would be hampered by some therapy‐sensitive mechanisms, such as PD‐1^+^ and Tregs, but not by CTLA‐4^+^ or determined inhibitory cytokines, that after chemoimmunotherapy would lead to complete tumor elimination. Importantly, we were able to characterize this phenotype through peripheral blood, and moreover, to identify some biomarkers that could be useful as baseline biomarkers for CPR.

Most of these differences for pretreatment biomarkers are lost posttreatment, probably due to treatment effect, that is able to modulate the immune cell repertoire. The main cell‐related biomarker associated with CPR after treatment is CD19 expression. CD19 is a classic marker for B‐cell identification, but also plays a key role as a coactivator for B‐cell function and proliferation.^53^ Elevated levels of this marker may be associated with increased humoral response and better clinical outcomes, because tertiary lymphoid structures have been associated with favorable prognosis.^13^ In fact, we found a correlation between CD19 expression and other immune cells, such as CD4^+^PD‐1^+^ and Tregs and CD56^+^ T cells, similar to previous results.^14^, ^54^

Although neoadjuvant chemotherapy for cancer has historically been considered immunosuppressive, we see that patients maintain general lymphocyte populations during treatment, similar to other studies evaluating monotherapy with anti‐PD‐1.^32^ In our study, neoadjuvant chemoimmunotherapy modified many subpopulations of lymphocytes and monocytes in peripheral blood, as well as more than 50 cytokines and soluble factors in plasma. Additionally, to general changes that occurred in all patients, our results show that some changes were exclusive of patients who achieved CPR or non‐CPR. This once again reinforces that they are distinct biological entities and the potential role of these changes as biomarkers of pathological response.

This study has several limitations such as the limited number of patients included in this analysis or the lack of control arm to discern between prognostic and predictive biomarkers. Additionally, the scarcity of disease progression events or deaths in these patients,^17^ precluded the association of these parameters to patient's survival.

To conclude, this is the first study focused on the composition and phenotypic characteristics of PBMCs in NSCLC patients treated with neoadjuvant chemoimmunotherapy. Patients achieving CPR seem to have a characteristic peripheral blood immune status at diagnosis that allows the identification of baseline blood‐based biomarkers associated to CPR. Additionally, CPR patients also showed a distinctive modification of their immune status as a consequence of neoadjuvant treatment with a different profile prior surgery. Further studies using larger cohorts of patients and including a control arm are warranted to validate these biomarkers in the neoadjuvant chemoimmunotherapy setting.

## CONFLICT OF INTEREST

Ernest Nadal reports personal fees from Bristol Myers Squibb, Merck Sharpe & Dohme, AstraZeneca, Lilly, Amgen, and Boehringer Ingelheim, and grants and personal fees from Roche and Pfizer, outside the submitted work. Amelia Insa reports personal fees from Bristol, BOERINGHER, MSD, PFIZER, Roche, and ASTRA ZENECA, outside the submitted work. María del Rosario García Campelo reports personal fees from BMS, MSD, Roche, Pfizer, and AstraZeneca, outside the submitted work. Manuel Dómine reports personal fees from Astra‐Zeneca, BMS, Boehringer Ingelheim, MSD, Pfizer, and Roche, outside the submitted work. Margarita Majem reports grants and personal fees from BMS, personal fees and nonfinancial support from MSD, BOEHRINGER INGELHEIM, ASTRA ZENENCA, ROCHE, and personal fees from KYOWA KYRIN and PIERRE FABRE, outside the submitted work. Delvys Rodríguez‐Abreu reports grants and personal fees from Bristol Myers Squibb, personal fees from GENENTECH/ROCHE, MSD, ASTRA ZENECA, BOEHRINGER INGELHEIM, Novartis, and Lilly, outside the submitted work. Alex Martínez‐Martí reports personal fees and nonfinancial support from Bristol Myers Squibb, F. Hoffmann La Roche AG, Merck Sharp & Dohme, Pfizer, Boehringer Ingelheim, MSD Oncology, and AstraZeneca, outside the submitted work. Javier De Castro Carpeño reports personal fees from Astra Zeneca, Boehringer Ingelheim, Merck Sharp and Dohme, Hoffmann‐la Roche, Bristol Myers Squibb, Takeda, Pfizer, and Novartis, outside the submitted work. Edel Del Barco reports nonfinancial support from ROCHE, BMS, PFIZER, ASTRA‐ZENECA, and MERCK, during the conduct of the study. Isidoro Barneto Aranda reports consulting or advisory board for Bristol Myers, Takeda, Roche, Astra Zeneca, and Behringer Inngelheim. Santiago Viteri reports personal fees and nonfinancial support from BMS, ROCHE, personal fees from MSD and ABBVIE, and nonfinancial support from OSE IMMUNOTHERAPEUTICS and MERCK SERONO, outside the submitted work. Bartomeu Massuti reports grants and personal fees from Roche, and personal fees and other from BMS, Takeda, MSD, and Boehringer, outside the submitted work. Cara Haymaker participated in scientific advisory board from Briacell. Ignacio I. Wistuba reports grants and personal fees from Genentech/Roche, Bayer, Bristol Myers Squibb, AstraZeneca/Medimmune, Pfizer, HTG Molecular, Merck, Guardant Health, and personal fees from GlaxoSmithKline, MSD, and Oncocyte, and grants from Oncoplex, DepArray, Adaptive, Adaptimmune, EMD Serono, Takeda, Amgen, Johnson & Johnson, Karus, Iovance, 4D, Novartis, and Akoya, outside the submitted work. Atocha Romero reports personal fees from Boehringer Ingelheim, outside the submitted work. Mariano Provencio reports grants, personal fees, and nonfinancial support from BMS, ROCHE, ASTRAZENECA, and personal fees from MSD and TAKEDA, outside the submitted work. Raquel Laza‐Briviesca, Alberto Cruz‐Bermúdez, Gerardo Huidobro, Manuel Cobo, Guillermo López Vivanco, Reyes Bernabé Caro, Nuria Viñolas, Marta Casarrubios, Belén Sierra‐Rodero, Carlos Tarín, Aránzazu García‐Grande, and Fernando Franco declare no conflict of interest.

## AUTHOR CONTRIBUTIONS

Alberto Cruz‐Bermúdez and Mariano Provencio conceived and designed the study. Ernest Nadal, Amelia Insa, María del Rosario García Campelo, Gerardo Huidobro, Manuel Dómine, Margarita Majem, Delvys Rodríguez‐Abreu, Alex Martínez‐Martí, Javier De Castro Carpeño, Manuel Cobo, Guillermo López Vivanco, Edel Del Barco, Reyes Bernabé Caro, Nuria Viñolas, Isidoro Barneto Aranda, Santiago Viteri, Bartomeu Massuti, Fernando Franco, and Mariano Provencio recruited and treated patients. Raquel Laza‐Briviesca, Marta Casarrubios, Belén Sierra‐Rodero, Alberto Cruz‐Bermúdez, Carlos Tarín, Atocha Romero and Aránzazu García‐Grande carried out the experiments and analyzed the data. Raquel Laza‐Briviesca, Alberto Cruz‐Bermúdez, Marta Casarrubios, Belén Sierra‐Rodero, Cara Haymaker, Ignacio I. Wistuba, Atocha Romero, and Mariano Provencio interpreted the data. All the authors have read and contributed to the final version of the manuscript and approved its submission for publication.

## Supporting information

SUPPORTING INFORMATION

SUPPORTING INFORMATION

## Data Availability

The data that support the findings of this study are available in the supplementary material of this article.
