# Supplementary material for: Blood biomarkers associated to complete pathological response on NSCLC patients treated with neoadjuvant chemoimmunotherapy included in NADIM clinical trial
Source: Clin Transl Med. 2021 Jul 19;11(7):e491. doi: 10.1002/ctm2.491 (PMC8288017; doi:10.1002/ctm2.491)
Supplement: Supplementary file 1 — SUPPORTING INFORMATION [file CTM2-11-e491-s001.pptx]

## Slide 1
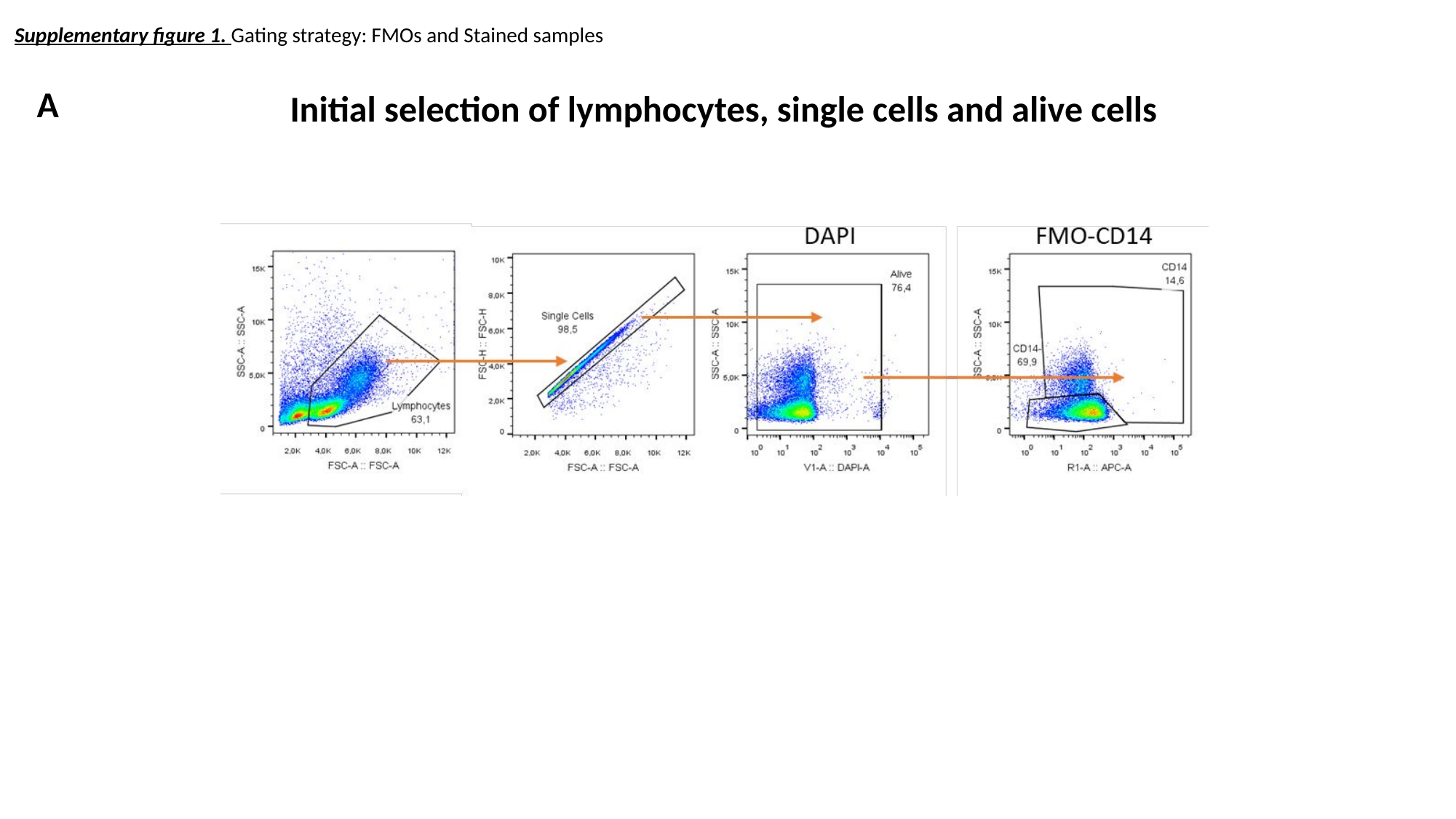

Supplementary figure 1. Gating strategy: FMOs and Stained samples
A
Initial selection of lymphocytes, single cells and alive cells

## Slide 2
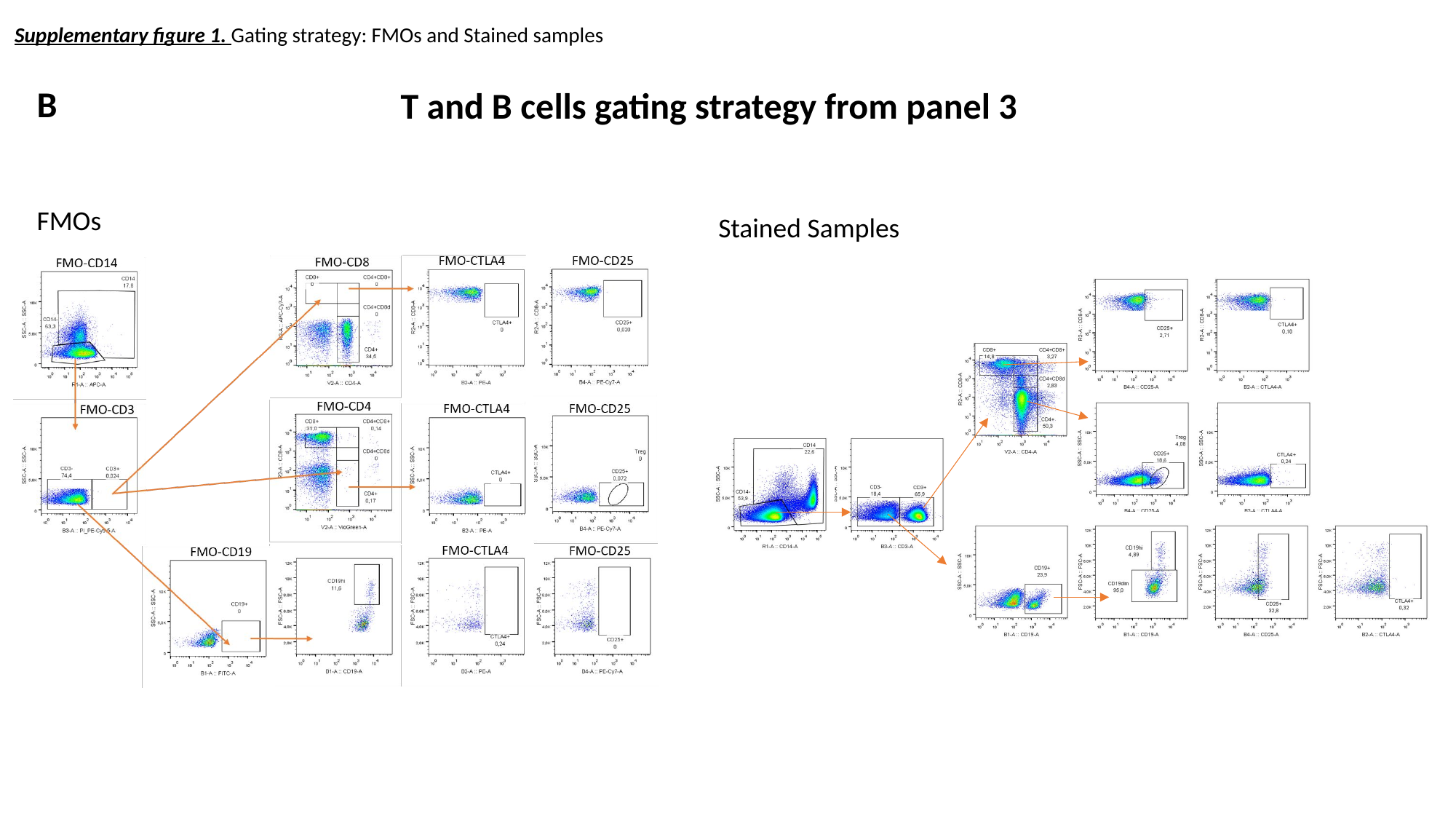

Supplementary figure 1. Gating strategy: FMOs and Stained samples
B
 T and B cells gating strategy from panel 3
FMOs
Stained Samples

## Slide 3
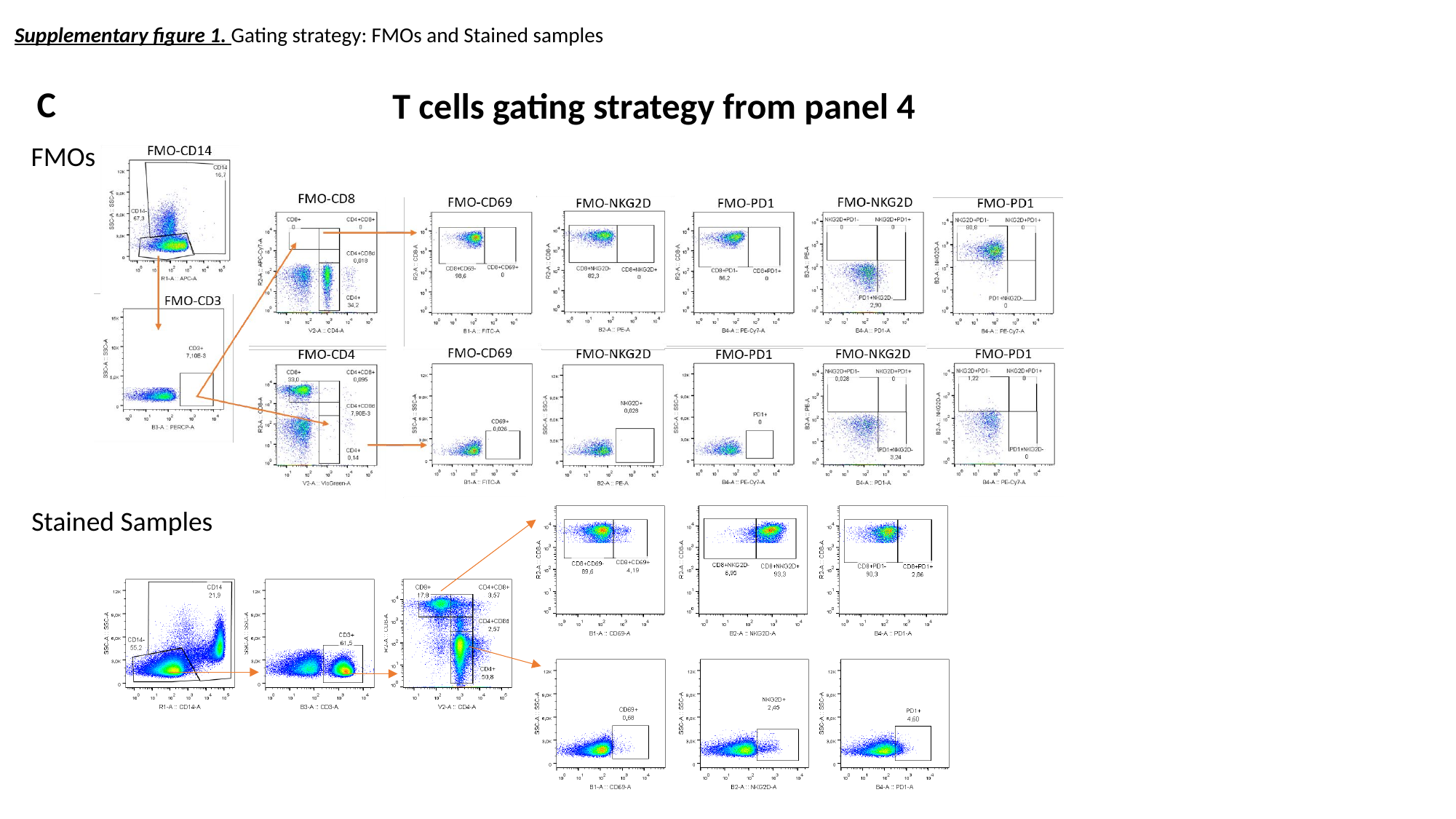

Supplementary figure 1. Gating strategy: FMOs and Stained samples
C
T cells gating strategy from panel 4
FMOs
Stained Samples

## Slide 4
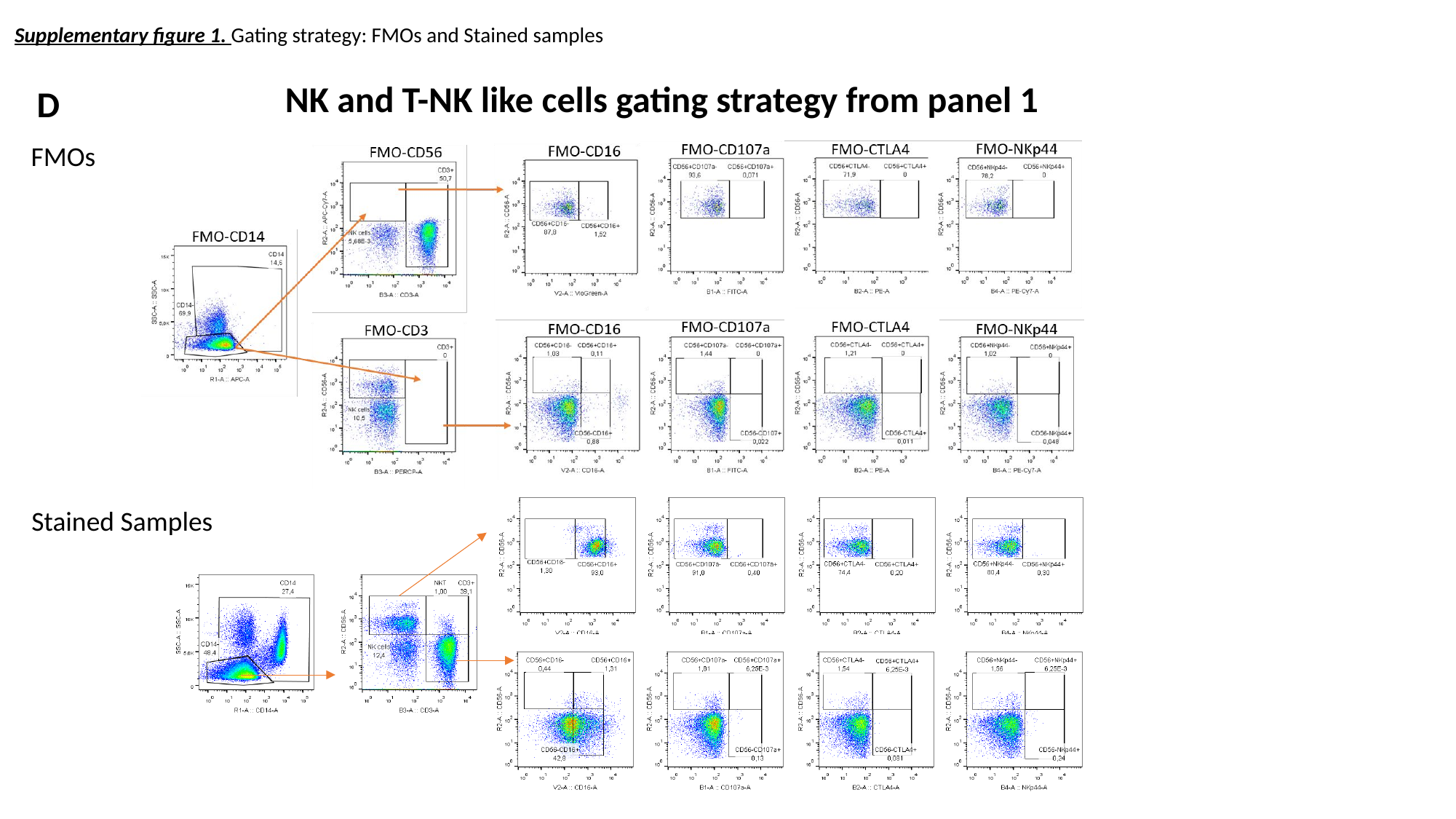

Supplementary figure 1. Gating strategy: FMOs and Stained samples
NK and T-NK like cells gating strategy from panel 1
D
FMOs
Stained Samples

## Slide 5
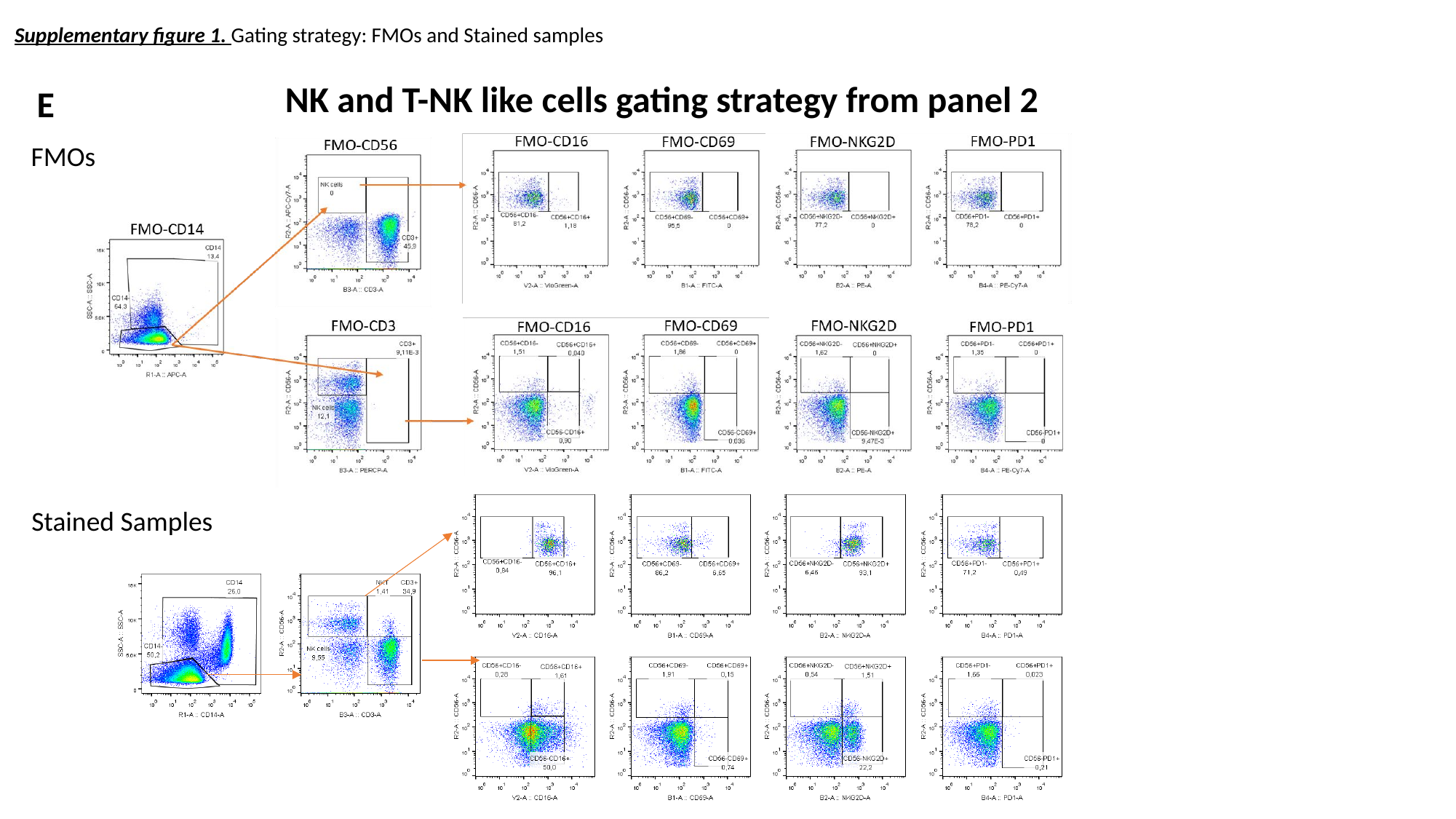

Supplementary figure 1. Gating strategy: FMOs and Stained samples
NK and T-NK like cells gating strategy from panel 2
E
FMOs
Stained Samples

## Slide 6
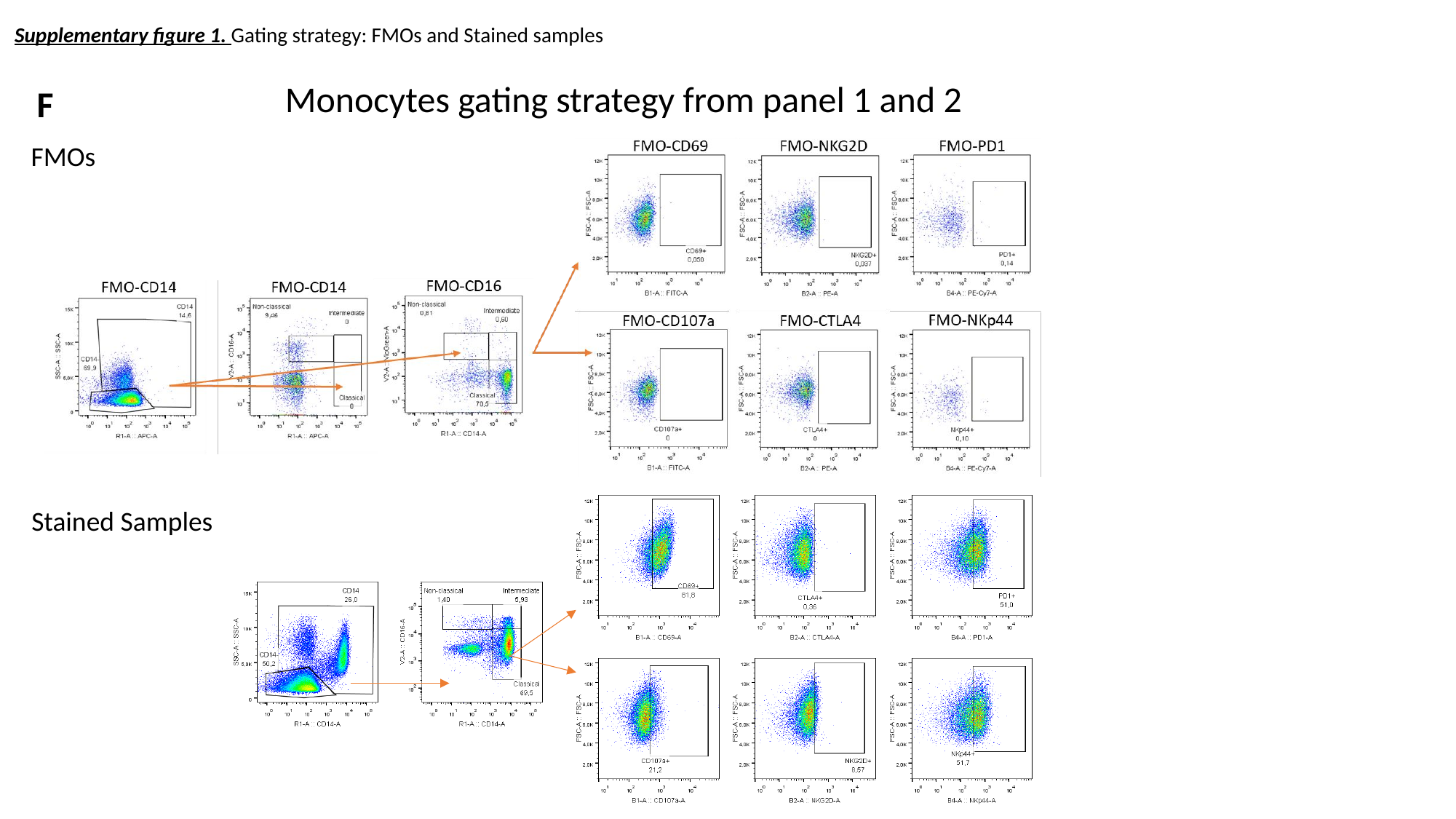

Supplementary figure 1. Gating strategy: FMOs and Stained samples
Monocytes gating strategy from panel 1 and 2
F
FMOs
Stained Samples

## Slide 7
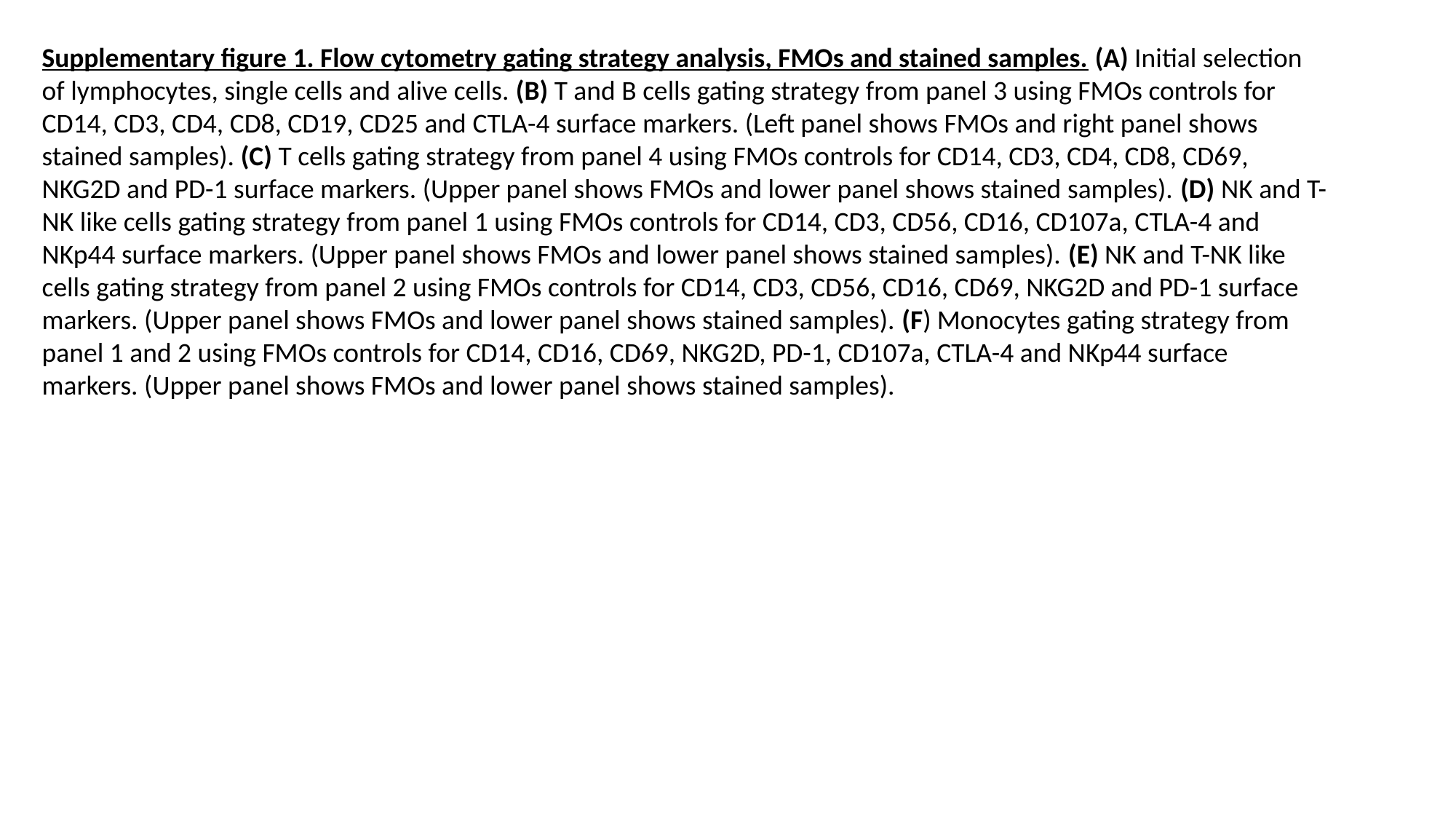

Supplementary figure 1. Flow cytometry gating strategy analysis, FMOs and stained samples. (A) Initial selection of lymphocytes, single cells and alive cells. (B) T and B cells gating strategy from panel 3 using FMOs controls for CD14, CD3, CD4, CD8, CD19, CD25 and CTLA-4 surface markers. (Left panel shows FMOs and right panel shows stained samples). (C) T cells gating strategy from panel 4 using FMOs controls for CD14, CD3, CD4, CD8, CD69, NKG2D and PD-1 surface markers. (Upper panel shows FMOs and lower panel shows stained samples). (D) NK and T-NK like cells gating strategy from panel 1 using FMOs controls for CD14, CD3, CD56, CD16, CD107a, CTLA-4 and NKp44 surface markers. (Upper panel shows FMOs and lower panel shows stained samples). (E) NK and T-NK like cells gating strategy from panel 2 using FMOs controls for CD14, CD3, CD56, CD16, CD69, NKG2D and PD-1 surface markers. (Upper panel shows FMOs and lower panel shows stained samples). (F) Monocytes gating strategy from panel 1 and 2 using FMOs controls for CD14, CD16, CD69, NKG2D, PD-1, CD107a, CTLA-4 and NKp44 surface markers. (Upper panel shows FMOs and lower panel shows stained samples).

## Slide 8
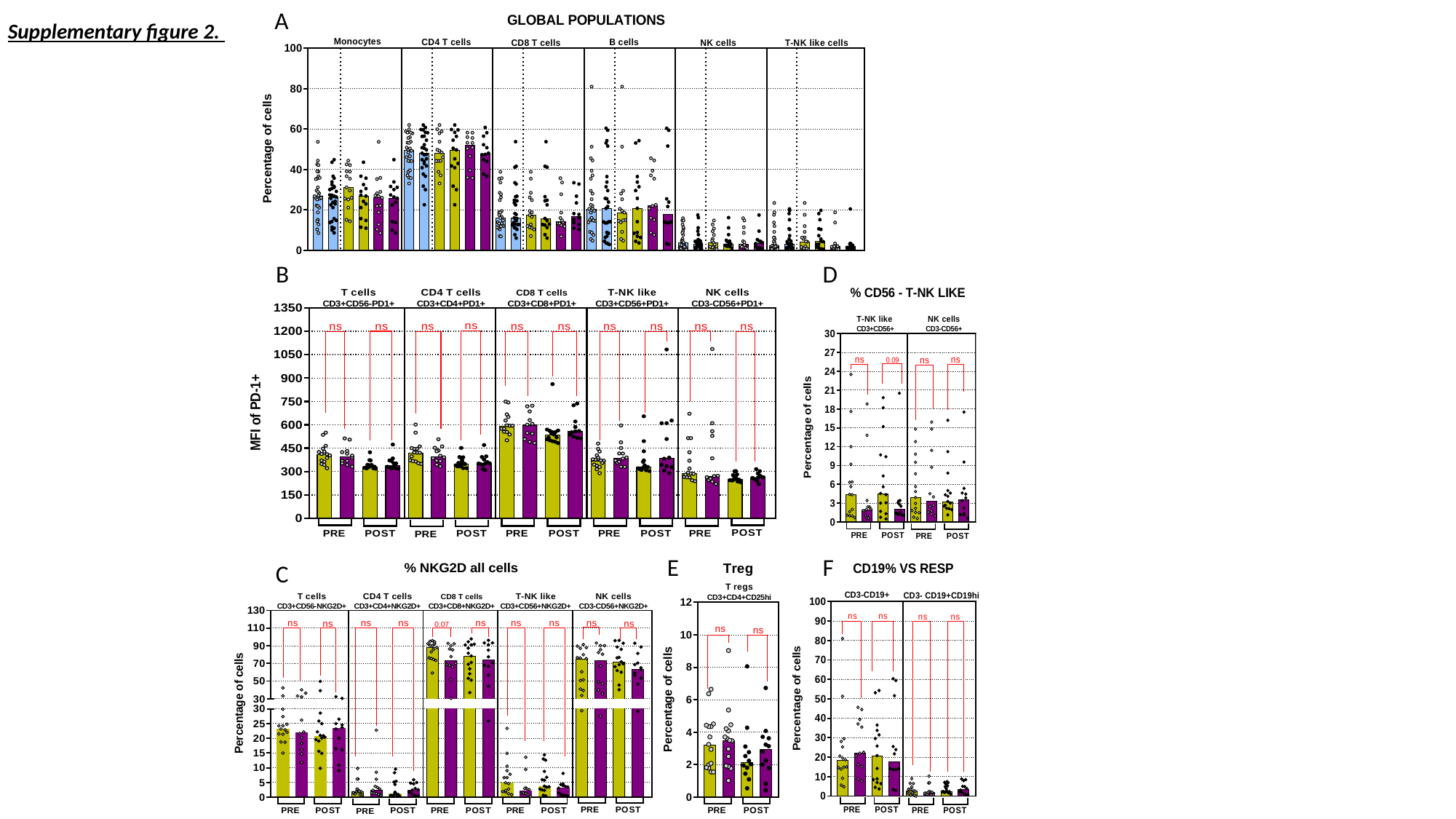

Supplementary figure 2.
A
B
D
E
F
C

## Slide 9
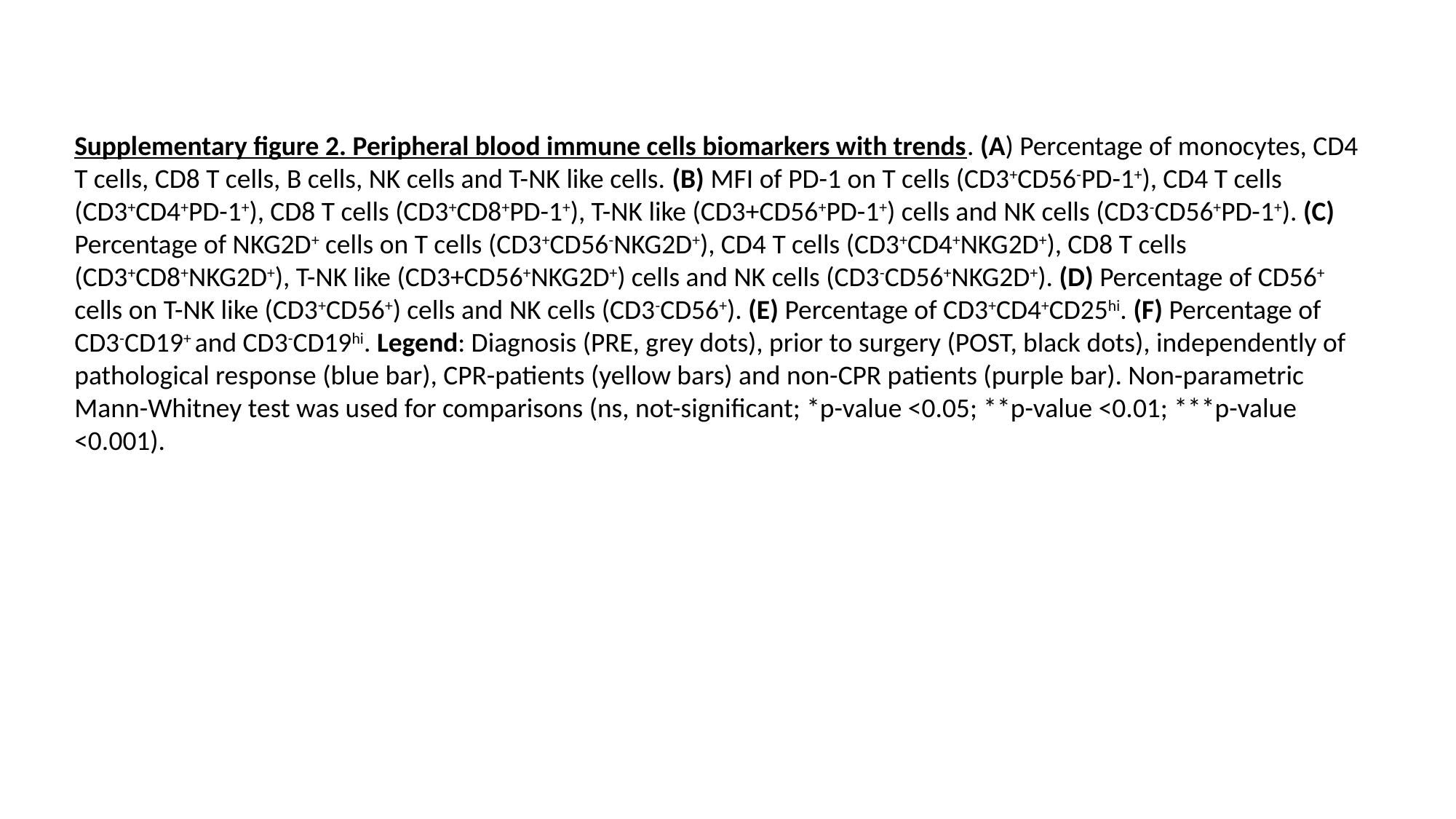

Supplementary figure 2. Peripheral blood immune cells biomarkers with trends. (A) Percentage of monocytes, CD4 T cells, CD8 T cells, B cells, NK cells and T-NK like cells. (B) MFI of PD-1 on T cells (CD3+CD56-PD-1+), CD4 T cells (CD3+CD4+PD-1+), CD8 T cells (CD3+CD8+PD-1+), T-NK like (CD3+CD56+PD-1+) cells and NK cells (CD3-CD56+PD-1+). (C) Percentage of NKG2D+ cells on T cells (CD3+CD56-NKG2D+), CD4 T cells (CD3+CD4+NKG2D+), CD8 T cells (CD3+CD8+NKG2D+), T-NK like (CD3+CD56+NKG2D+) cells and NK cells (CD3-CD56+NKG2D+). (D) Percentage of CD56+ cells on T-NK like (CD3+CD56+) cells and NK cells (CD3-CD56+). (E) Percentage of CD3+CD4+CD25hi. (F) Percentage of CD3-CD19+ and CD3-CD19hi. Legend: Diagnosis (PRE, grey dots), prior to surgery (POST, black dots), independently of pathological response (blue bar), CPR-patients (yellow bars) and non-CPR patients (purple bar). Non-parametric Mann-Whitney test was used for comparisons (ns, not-significant; *p-value <0.05; **p-value <0.01; ***p-value <0.001).

## Slide 10
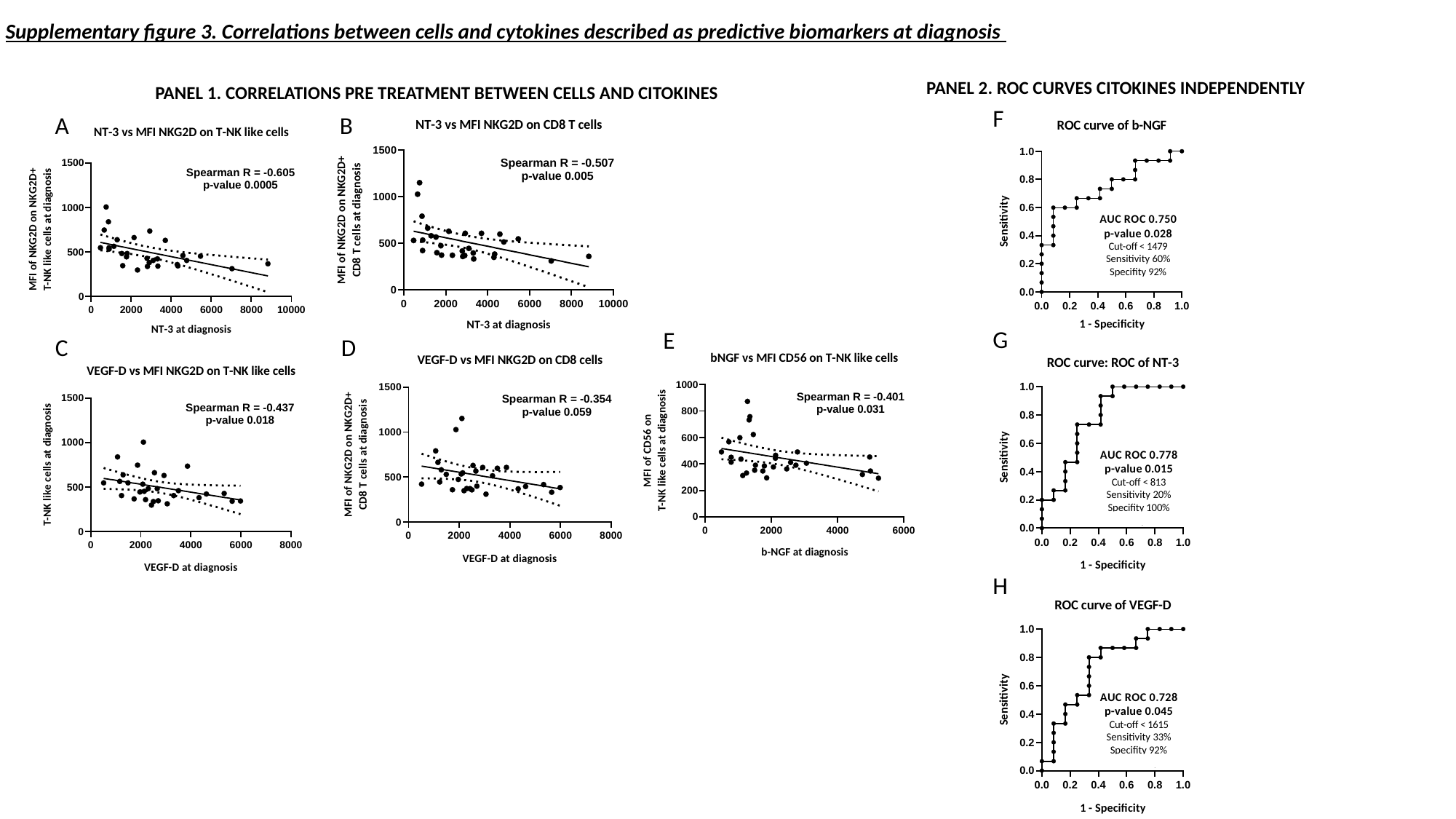

Supplementary figure 3. Correlations between cells and cytokines described as predictive biomarkers at diagnosis
PANEL 2. ROC CURVES CITOKINES INDEPENDENTLY
PANEL 1. CORRELATIONS PRE TREATMENT BETWEEN CELLS AND CITOKINES
F
A
B
G
E
D
C
H

## Slide 11
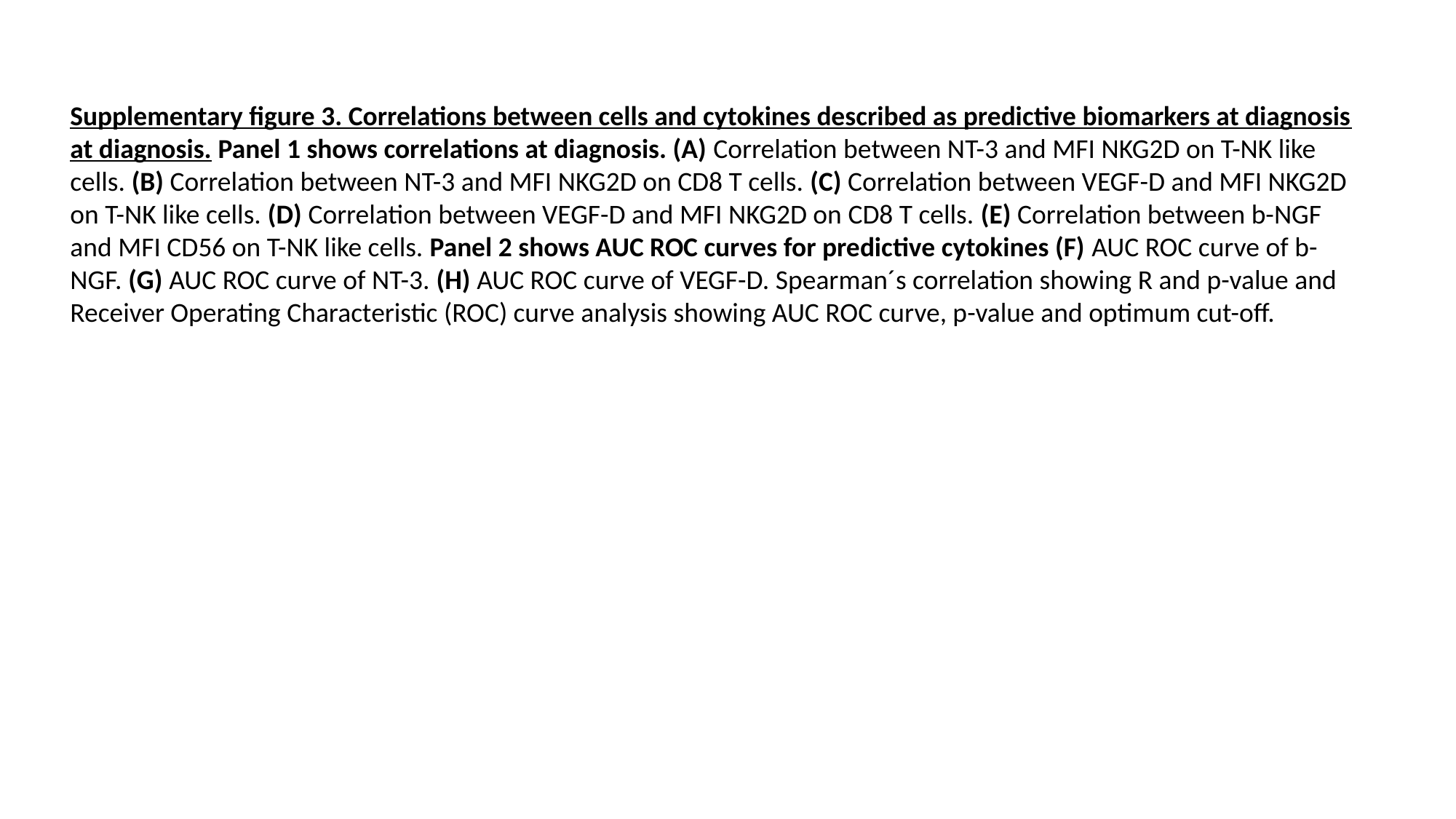

Supplementary figure 3. Correlations between cells and cytokines described as predictive biomarkers at diagnosis at diagnosis. Panel 1 shows correlations at diagnosis. (A) Correlation between NT-3 and MFI NKG2D on T-NK like cells. (B) Correlation between NT-3 and MFI NKG2D on CD8 T cells. (C) Correlation between VEGF-D and MFI NKG2D on T-NK like cells. (D) Correlation between VEGF-D and MFI NKG2D on CD8 T cells. (E) Correlation between b-NGF and MFI CD56 on T-NK like cells. Panel 2 shows AUC ROC curves for predictive cytokines (F) AUC ROC curve of b-NGF. (G) AUC ROC curve of NT-3. (H) AUC ROC curve of VEGF-D. Spearman´s correlation showing R and p-value and Receiver Operating Characteristic (ROC) curve analysis showing AUC ROC curve, p-value and optimum cut-off.

## Slide 12
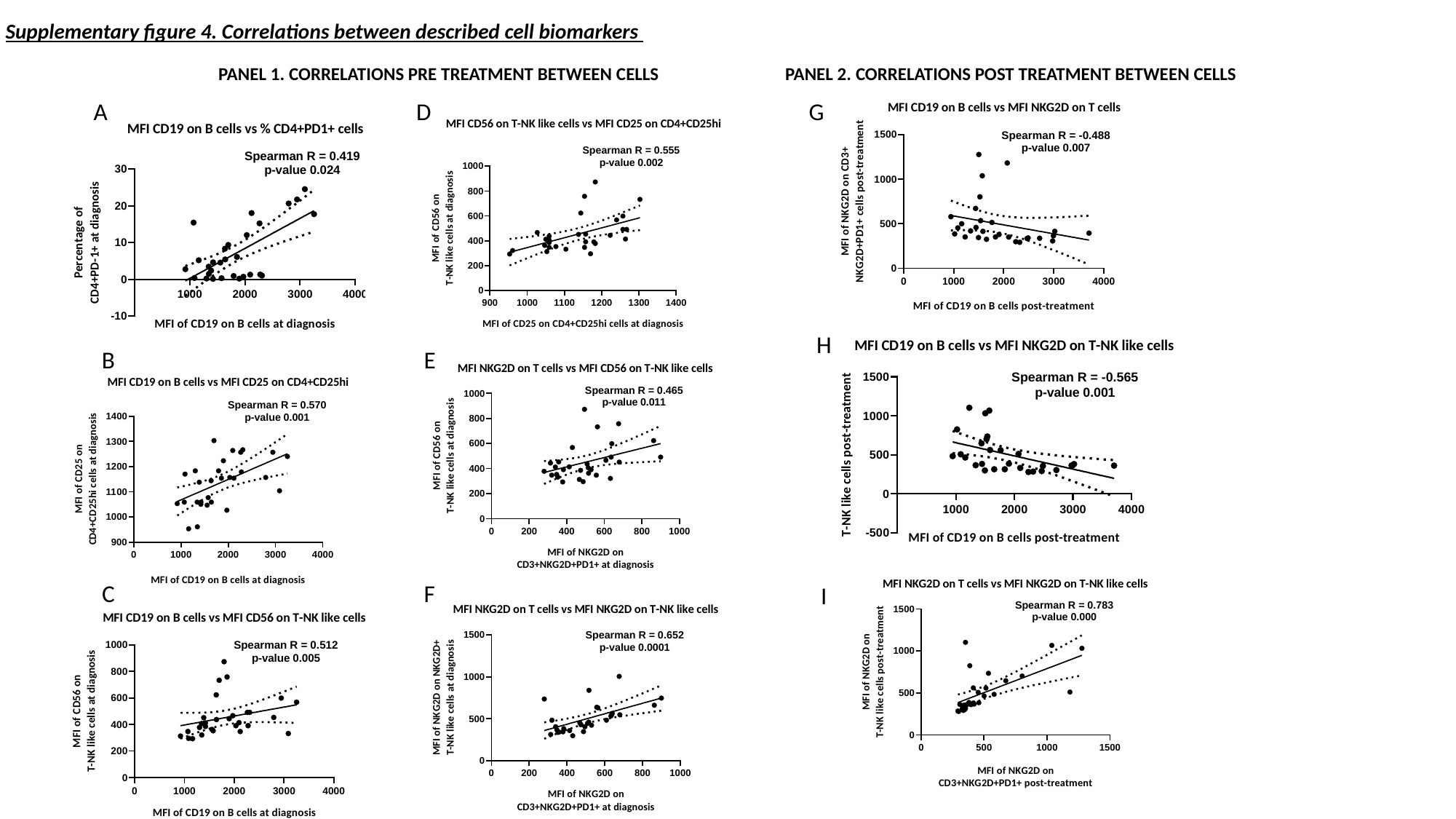

Supplementary figure 4. Correlations between described cell biomarkers
PANEL 2. CORRELATIONS POST TREATMENT BETWEEN CELLS
PANEL 1. CORRELATIONS PRE TREATMENT BETWEEN CELLS
A
D
G
H
B
E
C
F
I

## Slide 13
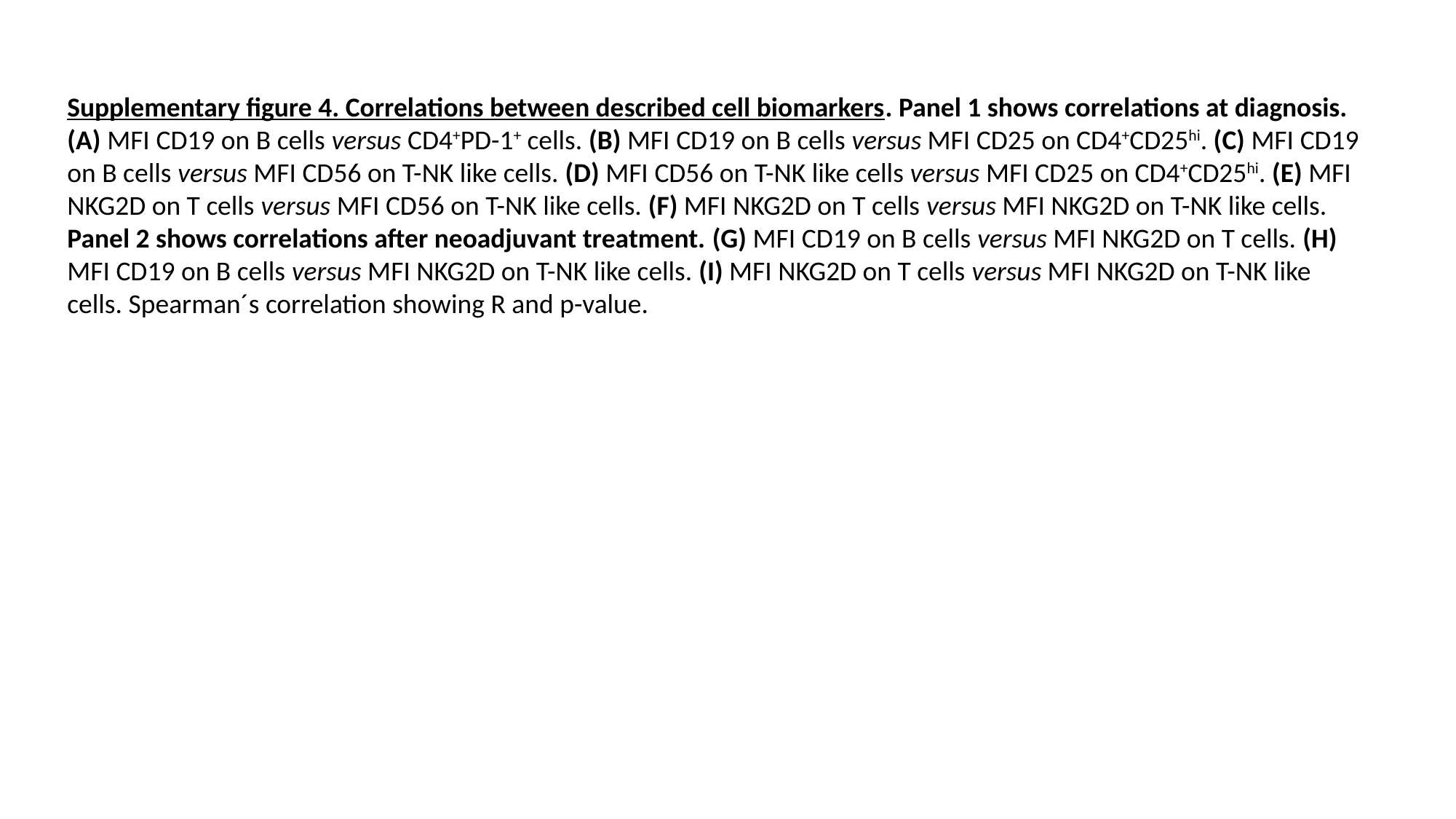

Supplementary figure 4. Correlations between described cell biomarkers. Panel 1 shows correlations at diagnosis. (A) MFI CD19 on B cells versus CD4+PD-1+ cells. (B) MFI CD19 on B cells versus MFI CD25 on CD4+CD25hi. (C) MFI CD19 on B cells versus MFI CD56 on T-NK like cells. (D) MFI CD56 on T-NK like cells versus MFI CD25 on CD4+CD25hi. (E) MFI NKG2D on T cells versus MFI CD56 on T-NK like cells. (F) MFI NKG2D on T cells versus MFI NKG2D on T-NK like cells. Panel 2 shows correlations after neoadjuvant treatment. (G) MFI CD19 on B cells versus MFI NKG2D on T cells. (H) MFI CD19 on B cells versus MFI NKG2D on T-NK like cells. (I) MFI NKG2D on T cells versus MFI NKG2D on T-NK like cells. Spearman´s correlation showing R and p-value.

## Slide 14
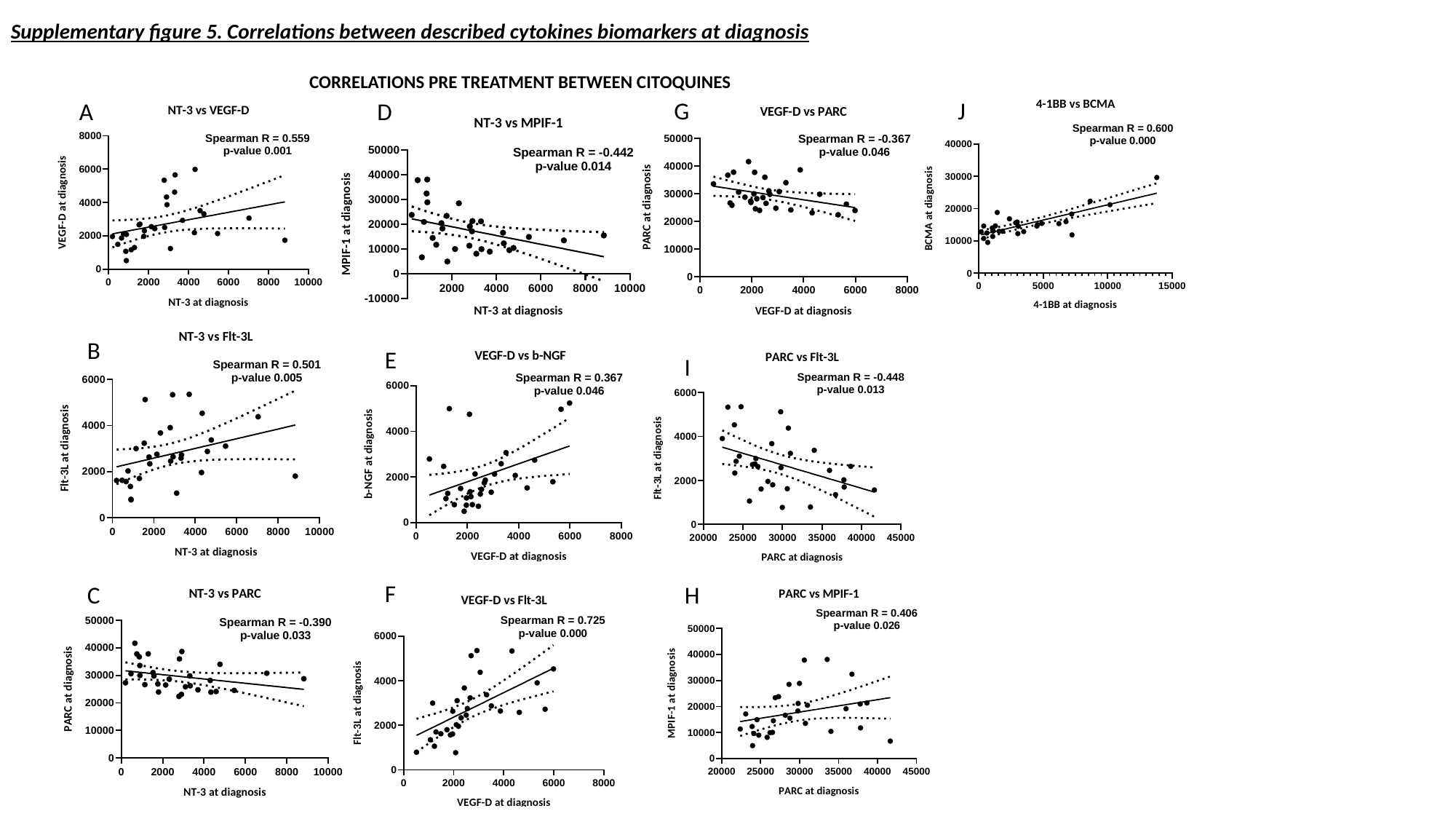

Supplementary figure 5. Correlations between described cytokines biomarkers at diagnosis
CORRELATIONS PRE TREATMENT BETWEEN CITOQUINES
G
J
A
D
B
E
I
F
C
H

## Slide 15
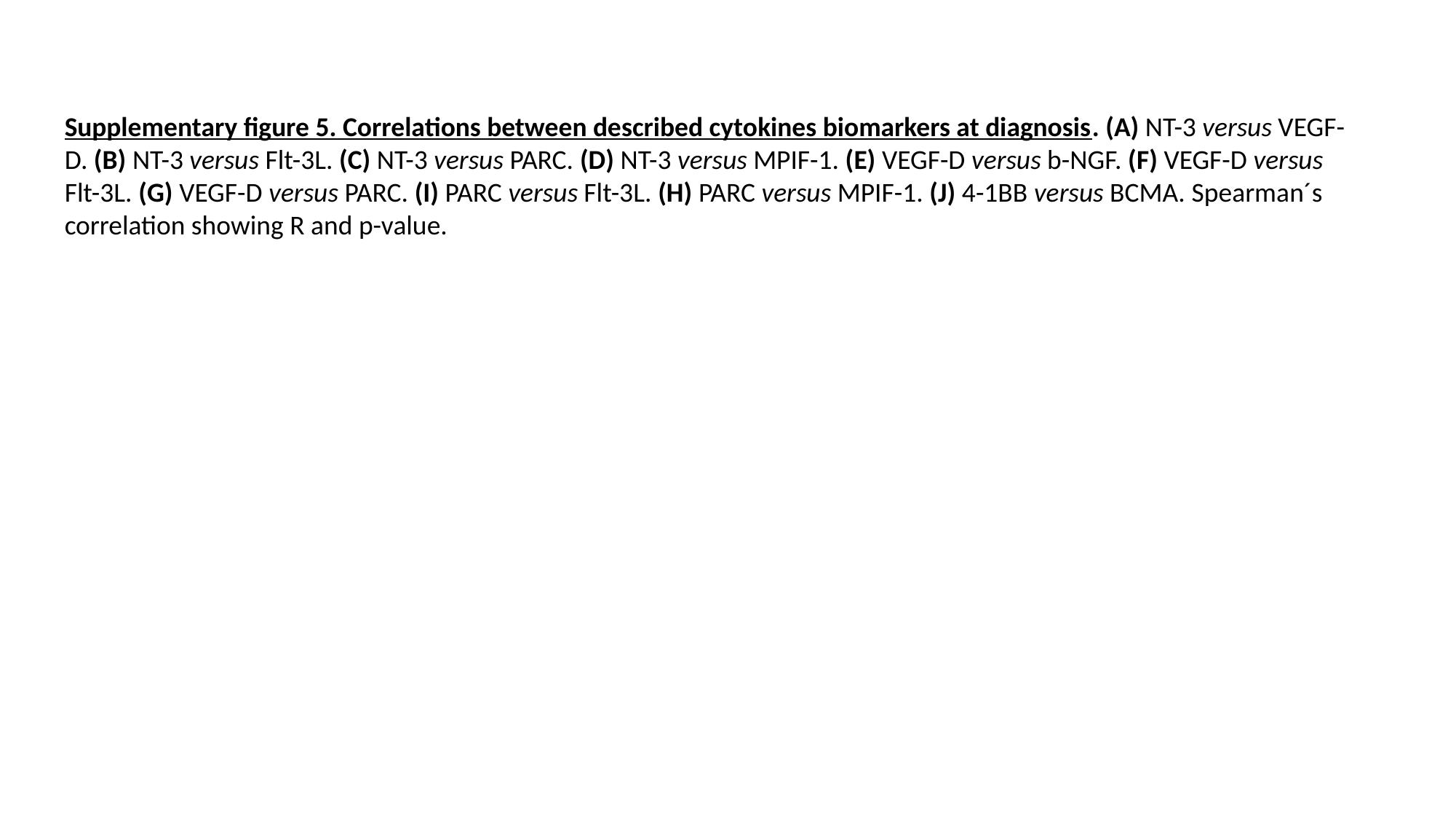

Supplementary figure 5. Correlations between described cytokines biomarkers at diagnosis. (A) NT-3 versus VEGF-D. (B) NT-3 versus Flt-3L. (C) NT-3 versus PARC. (D) NT-3 versus MPIF-1. (E) VEGF-D versus b-NGF. (F) VEGF-D versus Flt-3L. (G) VEGF-D versus PARC. (I) PARC versus Flt-3L. (H) PARC versus MPIF-1. (J) 4-1BB versus BCMA. Spearman´s correlation showing R and p-value.

## Slide 16
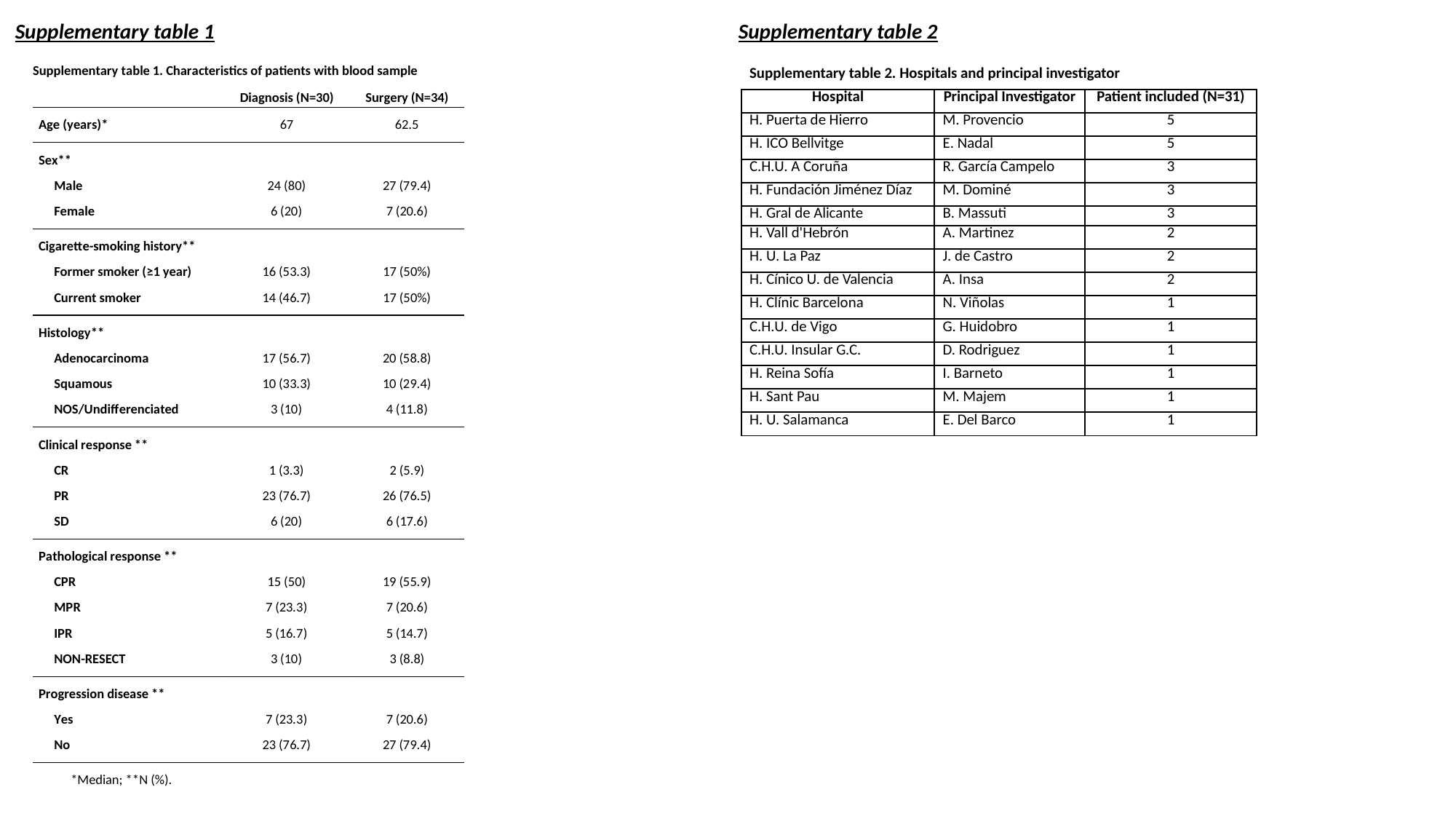

Supplementary table 1
Supplementary table 2
| Supplementary table 2. Hospitals and principal investigator | | |
| --- | --- | --- |
| Hospital | Principal Investigator | Patient included (N=31) |
| H. Puerta de Hierro | M. Provencio | 5 |
| H. ICO Bellvitge | E. Nadal | 5 |
| C.H.U. A Coruña | R. García Campelo | 3 |
| H. Fundación Jiménez Díaz | M. Dominé | 3 |
| H. Gral de Alicante | B. Massuti | 3 |
| H. Vall d'Hebrón | A. Martinez | 2 |
| H. U. La Paz | J. de Castro | 2 |
| H. Cínico U. de Valencia | A. Insa | 2 |
| H. Clínic Barcelona | N. Viñolas | 1 |
| C.H.U. de Vigo | G. Huidobro | 1 |
| C.H.U. Insular G.C. | D. Rodriguez | 1 |
| H. Reina Sofía | I. Barneto | 1 |
| H. Sant Pau | M. Majem | 1 |
| H. U. Salamanca | E. Del Barco | 1 |

## Slide 17
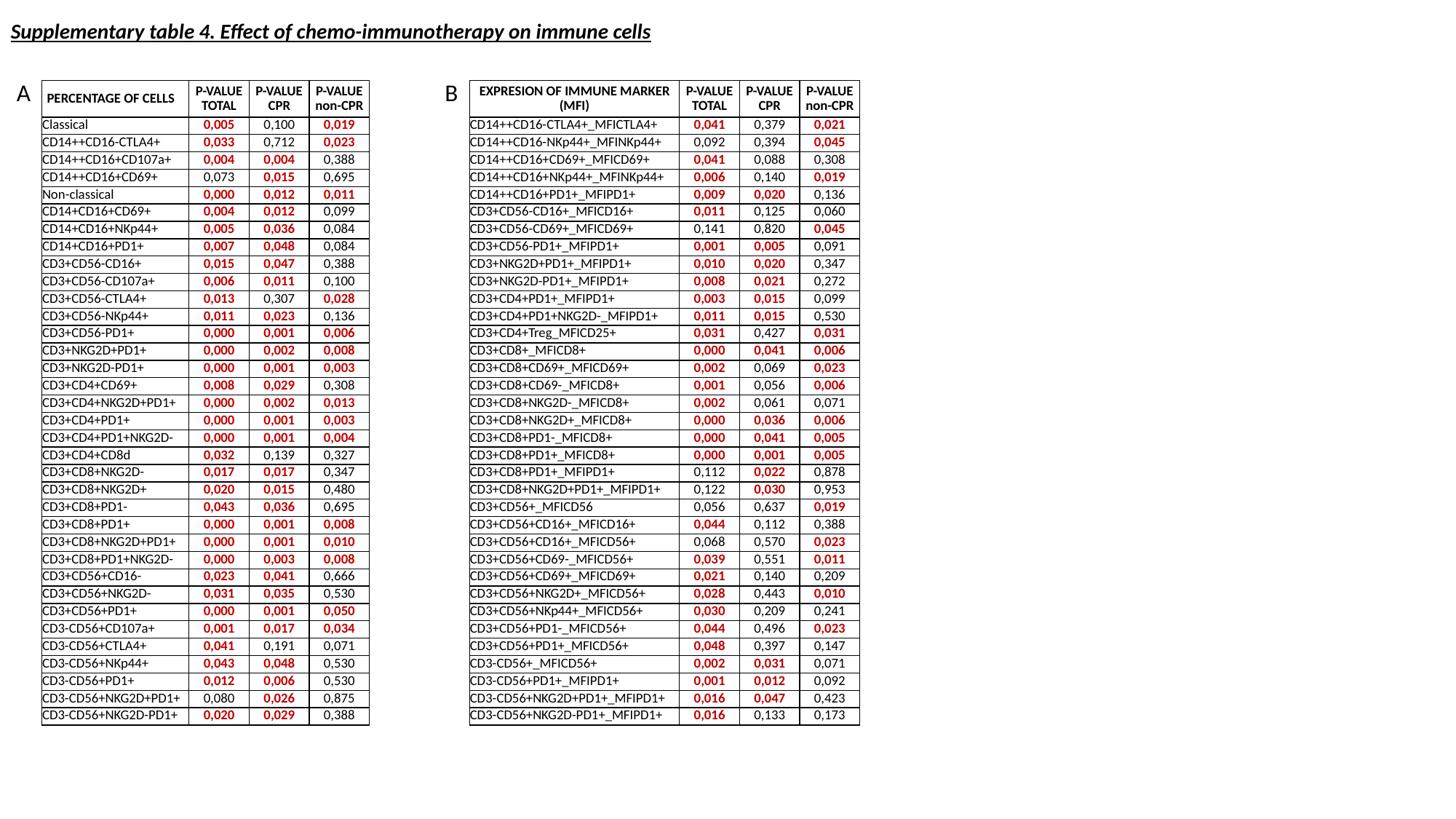

Supplementary table 4. Effect of chemo-immunotherapy on immune cells
A
B
| PERCENTAGE OF CELLS | P-VALUE TOTAL | P-VALUE CPR | P-VALUE non-CPR |
| --- | --- | --- | --- |
| Classical | 0,005 | 0,100 | 0,019 |
| CD14++CD16-CTLA4+ | 0,033 | 0,712 | 0,023 |
| CD14++CD16+CD107a+ | 0,004 | 0,004 | 0,388 |
| CD14++CD16+CD69+ | 0,073 | 0,015 | 0,695 |
| Non-classical | 0,000 | 0,012 | 0,011 |
| CD14+CD16+CD69+ | 0,004 | 0,012 | 0,099 |
| CD14+CD16+NKp44+ | 0,005 | 0,036 | 0,084 |
| CD14+CD16+PD1+ | 0,007 | 0,048 | 0,084 |
| CD3+CD56-CD16+ | 0,015 | 0,047 | 0,388 |
| CD3+CD56-CD107a+ | 0,006 | 0,011 | 0,100 |
| CD3+CD56-CTLA4+ | 0,013 | 0,307 | 0,028 |
| CD3+CD56-NKp44+ | 0,011 | 0,023 | 0,136 |
| CD3+CD56-PD1+ | 0,000 | 0,001 | 0,006 |
| CD3+NKG2D+PD1+ | 0,000 | 0,002 | 0,008 |
| CD3+NKG2D-PD1+ | 0,000 | 0,001 | 0,003 |
| CD3+CD4+CD69+ | 0,008 | 0,029 | 0,308 |
| CD3+CD4+NKG2D+PD1+ | 0,000 | 0,002 | 0,013 |
| CD3+CD4+PD1+ | 0,000 | 0,001 | 0,003 |
| CD3+CD4+PD1+NKG2D- | 0,000 | 0,001 | 0,004 |
| CD3+CD4+CD8d | 0,032 | 0,139 | 0,327 |
| CD3+CD8+NKG2D- | 0,017 | 0,017 | 0,347 |
| CD3+CD8+NKG2D+ | 0,020 | 0,015 | 0,480 |
| CD3+CD8+PD1- | 0,043 | 0,036 | 0,695 |
| CD3+CD8+PD1+ | 0,000 | 0,001 | 0,008 |
| CD3+CD8+NKG2D+PD1+ | 0,000 | 0,001 | 0,010 |
| CD3+CD8+PD1+NKG2D- | 0,000 | 0,003 | 0,008 |
| CD3+CD56+CD16- | 0,023 | 0,041 | 0,666 |
| CD3+CD56+NKG2D- | 0,031 | 0,035 | 0,530 |
| CD3+CD56+PD1+ | 0,000 | 0,001 | 0,050 |
| CD3-CD56+CD107a+ | 0,001 | 0,017 | 0,034 |
| CD3-CD56+CTLA4+ | 0,041 | 0,191 | 0,071 |
| CD3-CD56+NKp44+ | 0,043 | 0,048 | 0,530 |
| CD3-CD56+PD1+ | 0,012 | 0,006 | 0,530 |
| CD3-CD56+NKG2D+PD1+ | 0,080 | 0,026 | 0,875 |
| CD3-CD56+NKG2D-PD1+ | 0,020 | 0,029 | 0,388 |
| EXPRESION OF IMMUNE MARKER (MFI) | P-VALUE TOTAL | P-VALUE CPR | P-VALUE non-CPR |
| --- | --- | --- | --- |
| CD14++CD16-CTLA4+\_MFICTLA4+ | 0,041 | 0,379 | 0,021 |
| CD14++CD16-NKp44+\_MFINKp44+ | 0,092 | 0,394 | 0,045 |
| CD14++CD16+CD69+\_MFICD69+ | 0,041 | 0,088 | 0,308 |
| CD14++CD16+NKp44+\_MFINKp44+ | 0,006 | 0,140 | 0,019 |
| CD14++CD16+PD1+\_MFIPD1+ | 0,009 | 0,020 | 0,136 |
| CD3+CD56-CD16+\_MFICD16+ | 0,011 | 0,125 | 0,060 |
| CD3+CD56-CD69+\_MFICD69+ | 0,141 | 0,820 | 0,045 |
| CD3+CD56-PD1+\_MFIPD1+ | 0,001 | 0,005 | 0,091 |
| CD3+NKG2D+PD1+\_MFIPD1+ | 0,010 | 0,020 | 0,347 |
| CD3+NKG2D-PD1+\_MFIPD1+ | 0,008 | 0,021 | 0,272 |
| CD3+CD4+PD1+\_MFIPD1+ | 0,003 | 0,015 | 0,099 |
| CD3+CD4+PD1+NKG2D-\_MFIPD1+ | 0,011 | 0,015 | 0,530 |
| CD3+CD4+Treg\_MFICD25+ | 0,031 | 0,427 | 0,031 |
| CD3+CD8+\_MFICD8+ | 0,000 | 0,041 | 0,006 |
| CD3+CD8+CD69+\_MFICD69+ | 0,002 | 0,069 | 0,023 |
| CD3+CD8+CD69-\_MFICD8+ | 0,001 | 0,056 | 0,006 |
| CD3+CD8+NKG2D-\_MFICD8+ | 0,002 | 0,061 | 0,071 |
| CD3+CD8+NKG2D+\_MFICD8+ | 0,000 | 0,036 | 0,006 |
| CD3+CD8+PD1-\_MFICD8+ | 0,000 | 0,041 | 0,005 |
| CD3+CD8+PD1+\_MFICD8+ | 0,000 | 0,001 | 0,005 |
| CD3+CD8+PD1+\_MFIPD1+ | 0,112 | 0,022 | 0,878 |
| CD3+CD8+NKG2D+PD1+\_MFIPD1+ | 0,122 | 0,030 | 0,953 |
| CD3+CD56+\_MFICD56 | 0,056 | 0,637 | 0,019 |
| CD3+CD56+CD16+\_MFICD16+ | 0,044 | 0,112 | 0,388 |
| CD3+CD56+CD16+\_MFICD56+ | 0,068 | 0,570 | 0,023 |
| CD3+CD56+CD69-\_MFICD56+ | 0,039 | 0,551 | 0,011 |
| CD3+CD56+CD69+\_MFICD69+ | 0,021 | 0,140 | 0,209 |
| CD3+CD56+NKG2D+\_MFICD56+ | 0,028 | 0,443 | 0,010 |
| CD3+CD56+NKp44+\_MFICD56+ | 0,030 | 0,209 | 0,241 |
| CD3+CD56+PD1-\_MFICD56+ | 0,044 | 0,496 | 0,023 |
| CD3+CD56+PD1+\_MFICD56+ | 0,048 | 0,397 | 0,147 |
| CD3-CD56+\_MFICD56+ | 0,002 | 0,031 | 0,071 |
| CD3-CD56+PD1+\_MFIPD1+ | 0,001 | 0,012 | 0,092 |
| CD3-CD56+NKG2D+PD1+\_MFIPD1+ | 0,016 | 0,047 | 0,423 |
| CD3-CD56+NKG2D-PD1+\_MFIPD1+ | 0,016 | 0,133 | 0,173 |

## Slide 18
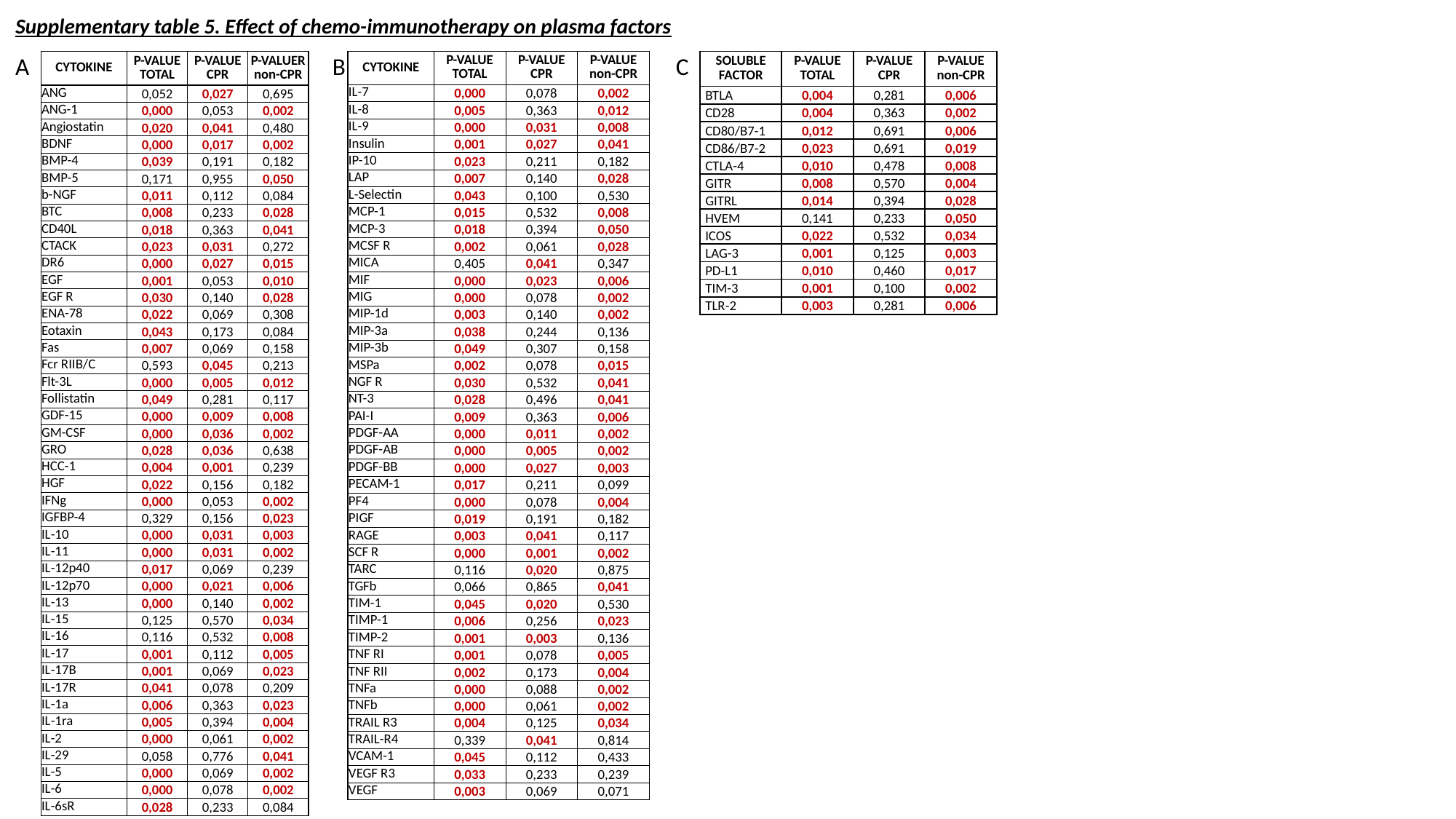

Supplementary table 5. Effect of chemo-immunotherapy on plasma factors
A
B
C
| CYTOKINE | P-VALUE TOTAL | P-VALUE CPR | P-VALUER non-CPR |
| --- | --- | --- | --- |
| ANG | 0,052 | 0,027 | 0,695 |
| ANG-1 | 0,000 | 0,053 | 0,002 |
| Angiostatin | 0,020 | 0,041 | 0,480 |
| BDNF | 0,000 | 0,017 | 0,002 |
| BMP-4 | 0,039 | 0,191 | 0,182 |
| BMP-5 | 0,171 | 0,955 | 0,050 |
| b-NGF | 0,011 | 0,112 | 0,084 |
| BTC | 0,008 | 0,233 | 0,028 |
| CD40L | 0,018 | 0,363 | 0,041 |
| CTACK | 0,023 | 0,031 | 0,272 |
| DR6 | 0,000 | 0,027 | 0,015 |
| EGF | 0,001 | 0,053 | 0,010 |
| EGF R | 0,030 | 0,140 | 0,028 |
| ENA-78 | 0,022 | 0,069 | 0,308 |
| Eotaxin | 0,043 | 0,173 | 0,084 |
| Fas | 0,007 | 0,069 | 0,158 |
| Fcr RIIB/C | 0,593 | 0,045 | 0,213 |
| Flt-3L | 0,000 | 0,005 | 0,012 |
| Follistatin | 0,049 | 0,281 | 0,117 |
| GDF-15 | 0,000 | 0,009 | 0,008 |
| GM-CSF | 0,000 | 0,036 | 0,002 |
| GRO | 0,028 | 0,036 | 0,638 |
| HCC-1 | 0,004 | 0,001 | 0,239 |
| HGF | 0,022 | 0,156 | 0,182 |
| IFNg | 0,000 | 0,053 | 0,002 |
| IGFBP-4 | 0,329 | 0,156 | 0,023 |
| IL-10 | 0,000 | 0,031 | 0,003 |
| IL-11 | 0,000 | 0,031 | 0,002 |
| IL-12p40 | 0,017 | 0,069 | 0,239 |
| IL-12p70 | 0,000 | 0,021 | 0,006 |
| IL-13 | 0,000 | 0,140 | 0,002 |
| IL-15 | 0,125 | 0,570 | 0,034 |
| IL-16 | 0,116 | 0,532 | 0,008 |
| IL-17 | 0,001 | 0,112 | 0,005 |
| IL-17B | 0,001 | 0,069 | 0,023 |
| IL-17R | 0,041 | 0,078 | 0,209 |
| IL-1a | 0,006 | 0,363 | 0,023 |
| IL-1ra | 0,005 | 0,394 | 0,004 |
| IL-2 | 0,000 | 0,061 | 0,002 |
| IL-29 | 0,058 | 0,776 | 0,041 |
| IL-5 | 0,000 | 0,069 | 0,002 |
| IL-6 | 0,000 | 0,078 | 0,002 |
| IL-6sR | 0,028 | 0,233 | 0,084 |
| CYTOKINE | P-VALUE TOTAL | P-VALUE CPR | P-VALUE non-CPR |
| --- | --- | --- | --- |
| IL-7 | 0,000 | 0,078 | 0,002 |
| IL-8 | 0,005 | 0,363 | 0,012 |
| IL-9 | 0,000 | 0,031 | 0,008 |
| Insulin | 0,001 | 0,027 | 0,041 |
| IP-10 | 0,023 | 0,211 | 0,182 |
| LAP | 0,007 | 0,140 | 0,028 |
| L-Selectin | 0,043 | 0,100 | 0,530 |
| MCP-1 | 0,015 | 0,532 | 0,008 |
| MCP-3 | 0,018 | 0,394 | 0,050 |
| MCSF R | 0,002 | 0,061 | 0,028 |
| MICA | 0,405 | 0,041 | 0,347 |
| MIF | 0,000 | 0,023 | 0,006 |
| MIG | 0,000 | 0,078 | 0,002 |
| MIP-1d | 0,003 | 0,140 | 0,002 |
| MIP-3a | 0,038 | 0,244 | 0,136 |
| MIP-3b | 0,049 | 0,307 | 0,158 |
| MSPa | 0,002 | 0,078 | 0,015 |
| NGF R | 0,030 | 0,532 | 0,041 |
| NT-3 | 0,028 | 0,496 | 0,041 |
| PAI-I | 0,009 | 0,363 | 0,006 |
| PDGF-AA | 0,000 | 0,011 | 0,002 |
| PDGF-AB | 0,000 | 0,005 | 0,002 |
| PDGF-BB | 0,000 | 0,027 | 0,003 |
| PECAM-1 | 0,017 | 0,211 | 0,099 |
| PF4 | 0,000 | 0,078 | 0,004 |
| PIGF | 0,019 | 0,191 | 0,182 |
| RAGE | 0,003 | 0,041 | 0,117 |
| SCF R | 0,000 | 0,001 | 0,002 |
| TARC | 0,116 | 0,020 | 0,875 |
| TGFb | 0,066 | 0,865 | 0,041 |
| TIM-1 | 0,045 | 0,020 | 0,530 |
| TIMP-1 | 0,006 | 0,256 | 0,023 |
| TIMP-2 | 0,001 | 0,003 | 0,136 |
| TNF RI | 0,001 | 0,078 | 0,005 |
| TNF RII | 0,002 | 0,173 | 0,004 |
| TNFa | 0,000 | 0,088 | 0,002 |
| TNFb | 0,000 | 0,061 | 0,002 |
| TRAIL R3 | 0,004 | 0,125 | 0,034 |
| TRAIL-R4 | 0,339 | 0,041 | 0,814 |
| VCAM-1 | 0,045 | 0,112 | 0,433 |
| VEGF R3 | 0,033 | 0,233 | 0,239 |
| VEGF | 0,003 | 0,069 | 0,071 |
| SOLUBLE FACTOR | P-VALUE TOTAL | P-VALUE CPR | P-VALUE non-CPR |
| --- | --- | --- | --- |
| BTLA | 0,004 | 0,281 | 0,006 |
| CD28 | 0,004 | 0,363 | 0,002 |
| CD80/B7-1 | 0,012 | 0,691 | 0,006 |
| CD86/B7-2 | 0,023 | 0,691 | 0,019 |
| CTLA-4 | 0,010 | 0,478 | 0,008 |
| GITR | 0,008 | 0,570 | 0,004 |
| GITRL | 0,014 | 0,394 | 0,028 |
| HVEM | 0,141 | 0,233 | 0,050 |
| ICOS | 0,022 | 0,532 | 0,034 |
| LAG-3 | 0,001 | 0,125 | 0,003 |
| PD-L1 | 0,010 | 0,460 | 0,017 |
| TIM-3 | 0,001 | 0,100 | 0,002 |
| TLR-2 | 0,003 | 0,281 | 0,006 |

## Slide 19
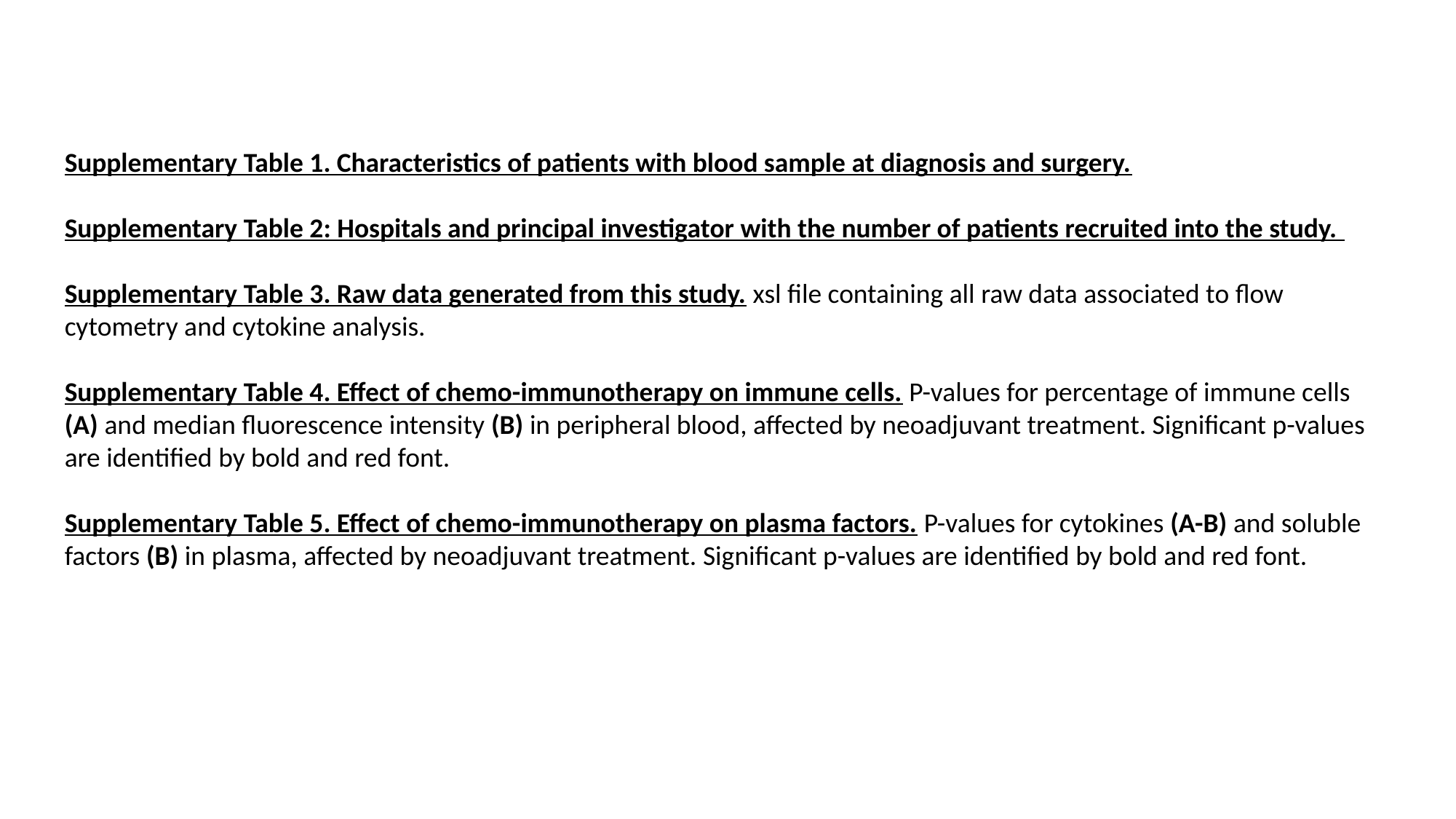

Supplementary Table 1. Characteristics of patients with blood sample at diagnosis and surgery.
Supplementary Table 2: Hospitals and principal investigator with the number of patients recruited into the study.
Supplementary Table 3. Raw data generated from this study. xsl file containing all raw data associated to flow cytometry and cytokine analysis.
Supplementary Table 4. Effect of chemo-immunotherapy on immune cells. P-values for percentage of immune cells (A) and median fluorescence intensity (B) in peripheral blood, affected by neoadjuvant treatment. Significant p-values are identified by bold and red font.
Supplementary Table 5. Effect of chemo-immunotherapy on plasma factors. P-values for cytokines (A-B) and soluble factors (B) in plasma, affected by neoadjuvant treatment. Significant p-values are identified by bold and red font.
